# Health-related quality of life in patients with advanced soft tissue sarcoma receiving first-line palliative chemotherapy (HOLISTIC): longitudinal results from a prospective, observational cohort study

**DOI:** 10.1016/j.eclinm.2025.103561

**Published:** 2025-10-16

**Authors:** Evelyne Roets, Eugenie Younger, Robin L. Jones, Dide den Hollander, Leyla Azarang, Ingrid M.E. Desar, Robin J. Young, Astrid W. Oosten, Jacco J. de Haan, Hans Gelderblom, Neeltje Steeghs, Olga Husson, Winette T.A. van der Graaf

**Affiliations:** a Department of Medical Oncology, The Netherlands Cancer Institute, Plesmanlaan 121, 1066 CX, Amsterdam, the Netherlands; b Sarcoma Unit, Royal Marsden NHS Foundation Trust, Fulham Road, SW3 6JJ, London, UK; c Division of Clinical Studies, Institute of Cancer Research, London, UK; d Department of Medical Oncology, Radboud University Medical Centre, Nijmegen, the Netherlands; e Academic Unit of Clinical Oncology, The University of Sheffield, Sheffield, UK; f Department of Medical Oncology, Erasmus MC Cancer Institute, Erasmus University Medical Center, Rotterdam, the Netherlands; g Department of Medical Oncology, University of Groningen, University Medical Center Groningen, Groningen, the Netherlands; h Department of Medical Oncology, Leiden University Medical Center, Leiden, the Netherlands; i Department of Surgical Oncology, ErasmusMC Cancer Institute, Erasmus University Medical Center, Doctor Molewaterplein 40, 3015 GD, Rotterdam, the Netherlands; j Department of Public Health, Erasmus MC Cancer Institute, Erasmus University Medical Center, 3015 GD, Rotterdam, the Netherlands

**Keywords:** Sarcomas, Quality of life, Global health, Systemic therapy

## Abstract

**Background:**

Previous research suggests that health-related quality of life (HRQoL) in patients with advanced soft tissue sarcoma (STS) is often severely impacted, but data on longitudinal changes during chemotherapy are lacking. We aimed to address this knowledge gap.

**Methods:**

This prospective, observational cohort study (HOLISTIC) assessed HRQoL in patients with advanced STS during first-line palliative chemotherapy, focusing on Global Health Scores (GHS) change after 4 cycles (T4). Eligible patients were recruited from five centres in the Netherlands (n = 63) and two centres in the United Kingdom (UK, n = 72). Clinical and sociodemographic data were collected, and HRQoL was measured using the EORTC QLQ-C30 at baseline (T0) and before each cycle. The primary outcome of this study was the change in GHS on the EORTC QLQ-C30 between baseline and after 4 cycles of first-line palliative chemotherapy. Changes in GHS were tested using paired sample t-tests and linear mixed-effects models (LME). For patients who did not complete 4 cycles, the last score post baseline (i.e. T3, T2) was used (i.e. T4/final). This study is registered with ClinicalTrials.gov, NCT03621332.

**Findings:**

Between March 2018 and March 2020, 137 patients from the UK (n = 72) and the Netherlands (n = 65) were enrolled. Two patients never started chemotherapy and were excluded. Of the remaining 135 patients, 60 (44%) had at least 4 cycles of chemotherapy and 91 (68%) completed the questionnaire at T0 together with at least the questionnaire at T2, T3 and/or T4. Mean GHS significantly deteriorated from 68.2 (T0) to 60.7 (T4/final) with a mean difference between T0 and T4/final of −11.3 points (95% CI 7.6–15.0, p < 0.001). GHS worsened both in patients with partial response/stable disease (−12.3 points, 95% CI 7.9–16.8, p < 0.001) and in patients with progressive disease (−10.9 points, 95% CI 2.3–19.6, p = 0.015). Baseline GHS were lower (i.e. worse) for patients with ECOG PS 1–2 (77.1 vs 84.7 [ECOG PS 0], p = 0.023), patients from the UK (77.4 vs 84.7 [NL], p = 0.008) and patients with anaemia (78.6 vs 84.7 [no anaemia], p = 0.040). The decline in GHS over time was more pronounced in patients with ECOG PS 0 (−4.7 points per cycle) compared to those with ECOG PS 1 or 2 (−1.4 points per cycle, (p = 0.014).

**Interpretation:**

First-line palliative chemotherapy in advanced STS is associated with a significant decrease in GHS, irrespective of tumour response. These results emphasise the importance of integrating patient-reported outcomes (PROs) in clinical trials and routine care, and may enable informed decision making by patients with advanced STS starting palliative chemotherapy. Future research should explore implementing PROs in practice, using them to guide treatment, and how chemotherapy vs disease progression affects QoL.

**Funding:**

10.13039/100004312Eli Lilly and Company.

**Research in context**

**Evidence before this study**

We searched PubMed for studies on health-related quality of life (HRQoL) in advanced soft tissue sarcoma (STS) patients receiving first-line palliative chemotherapy, using terms including “advanced soft tissue sarcoma”, “quality of life” and “chemotherapy”. The search included all languages and covered studies published from database inception to February 2025. Previous research suggests that HRQoL in patients with advanced STS is often severely impacted, but data on longitudinal changes during chemotherapy are lacking.

**Added value of this study**

To the best of our knowledge, this study is the first prospective investigation of HRQoL changes during standard first-line palliative chemotherapy in advanced STS. By using robust statistical methods and by including patients both from the United Kingdom and the Netherlands, we provide a comprehensive understanding of evolution of Global Health Score (GHS) over time. The use of linear mixed-effects models has allowed identification of clinical and sociodemographic factors influencing HRQoL, filling a critical gap in the literature.

**Implications of all the available evidence**

These findings highlight the significant decline in HRQoL during chemotherapy, regardless of tumour response. Fitter patients (i.e. ECOG PS 0) are more likely to experience a faster decline in GHS. This information is crucial for shared decision-making, and for providing personalised supportive measures in a timely way. Next steps should include prospectively following PROs in a larger STS population with minimal missing data and performing joint analyses of QoL and survival to better understand the impact of chemotherapy vs disease progression on QoL.

## Introduction

Soft tissue sarcomas (STS) are a group of rare, heterogeneous tumours of mesenchymal origin. Approximately 10% of patients present with metastatic disease and about 50% of patients with initially localised tumours will eventually develop advanced disease.^1^ Palliative chemotherapy with anthracyclines based regimens, has been the standard first-line treatment since the 1970s.^2^^,^^3^ The net clinical treatment benefit is determined both by the effect of the treatment on overall survival (OS) and quality of life (QoL).^4^ Despite an improvement of survival of patients with metastatic STS over time, median OS for this patient population is only 18 months, underscoring the importance of Health-Related Quality of Life (HRQoL).^5^ Hence, the principal aim of palliative systemic therapy is improving not only Length of Life (LoL) but also QoL and therefore more knowledge about impact of chemotherapy on QOL is urgently needed.

Systemic therapy may negatively affect HRQoL due to toxicity,^6^, ^7^, ^8^, ^9^ but it may also cause stabilisation or improvement of specific symptoms (e.g. pain).^10^ Despite this, most phase II-III RCTs of systemic therapy for patients with advanced STS focus on (progression-free) survival outcomes, with few including HRQoL as an endpoint.^11^ A systematic review of RCTs in this setting found that only 35% (15/43) of RCTs included a PRO endpoint and only 10 of these RCTs reported PRO results in the manuscript. Furthermore, quality of PRO reporting was low. This is despite growing evidence that PROs are independent prognostic factors for OS across cancer populations and disease stages.^12^ Prospectively measured HRQoL data could help to assess whether palliative chemotherapy offers effective palliation, give more insight into patients’ needs and support treatment decisions.

The HOLISTIC study (Health-related quality Of Life In patients with advanced Soft TIssue sarcomas treated with Chemotherapy) evaluated HRQoL in patients with advanced STS treated with first-line palliative chemotherapy. Baseline results (i.e. before start of treatment; T0), showed that half of patients prioritised QoL above LoL (41%) or prioritised LoL and QoL equally (9%).^13^

Here, we present the results of HOLISTIC's primary endpoint: the change in EORTC-QLQ-C30 GHS after 4 cycles with first-line palliative chemotherapy in patients with advanced STS. Secondary endpoints included changes in other QoL domains.

## Methods

### Study design and ethics

Full details of the protocol are published elsewhere^13^^,^^14^ and the study protocol was prospectively registered with ClinicalTrials.gov (NCT03621332). Ethical approval was obtained in the UK (REC 17/NI/1097) and in the Netherlands (Radboud University Medical Centre and UMCG: 2018–4151, Erasmus Medical Centre: MEC-2018-1101, Leiden University Medical Centre: P18.179 P1a, Netherlands Cancer Institute: 2018-12-04 18.453). All patients provided informed consent (electronically or written) before participation. This study included patients aged ≥18 years, receiving first-line palliative chemotherapy for advanced STS in five sarcoma reference centres in the UK and five in the Netherlands. Advanced disease was defined as metastatic disease, or locally advanced disease not amenable to curative surgical resection. Eligible patients were recruited by a member of the sarcoma team at the participating centres between March 2018 and March 2020. To achieve 90% power at a two-sided 5% significance level, the protocol required 119 patients; with 10% added for potential dropouts, the target was 132. A total of 135 patients were recruited. Data were collected using the Patient-Reported Outcomes Following Initial treatment and Long-term Evaluation of Survivorship (PROFILES) registry.^15^ Participants completed questionnaires (online or paper/English or Dutch) before starting first-line chemotherapy (i.e. baseline) and at the beginning of each cycle of chemotherapy and 3-monthly during follow-up.

### Participant characteristics

The baseline questionnaire contained sociodemographic characteristics, among which age, sex (male/female; self-reported), relationship status, and education. Clinical characteristics including number of metastatic sites, histological subtype, site of primary disease, baseline Eastern Cooperative Oncology Group (ECOG) performance status (PS), chemotherapy regimen and baseline laboratory values, were extracted from the medical record. The latter included: anaemia (Hb < 13.0 g/L male, <11.5 g/L female), lymphocytopenia <1 × 10^9^/L, lactate dehydrogenase (LDH) > 250 U/L and hypoalbuminaemia <35 g/L. ECOG PS was dichotomised (0 vs 1–2), reflecting “good” vs “poor” performance status. Chemotherapy regimens were classified as monotherapy or combination regimens. Combination regimens included both the use of two cytotoxic agents or the combination of a cytotoxic agent with a monoclonal antibody or immune checkpoint inhibitor. The first response evaluation after starting palliative first-line systemic therapy was reported according to RECIST (version 1.1) and was dichotomised into two categories: stable disease (SD) or partial response (PR) vs progressive disease (PD).^16^

### Questionnaires and outcomes

HRQoL was measured using the EORTC QLQ-C30 questionnaire, which consists of 30 items, including five functional scales (physical, role, cognitive, emotional and social), three symptom scales (fatigue, pain, nausea and vomiting), six single item symptom measures (dyspnoea, loss of appetite, insomnia, constipation and diarrhoea), and a GH status scale.^17^ All EORTC QLQ-C30 scales were transformed to linear scores ranging from 0 to 100.^18^ A higher score on EORTC QLQ-C30 function domains and GH means better functioning, whereas a higher score on the symptom domains means more complaints.^19^

The primary outcome of this study was the change in GHS on the EORTC QLQ-C30 between baseline and after 4 cycles of first-line palliative chemotherapy. Secondary outcomes included changes in other EORTC QLQ-C30 symptom and function domains over time, and the association of sociodemographic and clinical characteristics, as well as radiological response, with changes in GHS and other HRQoL scores over time.

### Statistical analysis

Changes in EORTC QLQ-C30 scores were tested using a paired sample t-test from baseline to after 4 cycles (T4) with a two-sided 5% significance level. For patients who did not complete 4 cycles, the last score post baseline (i.e. T3, T2) was used (i.e. T4/final). Mean scores and standard error (SE) at each time point were graphically represented. The mean difference in GHS between T0 and T4/final was calculated using a paired sample t-test.

For GHS, a pre-specified sensitivity analysis was performed excluding patients who did not reach 4 cycles. For the EORTC QLQ-C30, the EORTC scoring manual guidelines were used for missing data.^17^ Guidelines published by Cocks et al. were used to determine clinically relevant differences: ≥10 points for GHS, ≥8 points for diarrhoea, nausea and vomiting, ≥9 points for cognitive functioning and dyspnoea, ≥11 points for social functioning, ≥13 points for insomnia, fatigue, constipation, pain, ≥14 points for physical functioning, appetite loss and ≥19 points for role functioning.^19^ To assess the effect of radiological response on GHS, the questionnaire closest to the response evaluation time point was used.

Linear mixed-effects (LME) models were used to assess EORTC QLQ-C30 scores at baseline and changes over time.^20^ We fitted LME models with random intercepts and random slopes for time at the participant level, to account for repeated measures and individual variability in both baseline scores and trajectories over time. This modelling approach estimates average trends over time while incorporating data from all patients, including those who did not complete all four treatment cycles. Sociodemographic and clinical factors were included as fixed effects to assess their association with HRQoL outcomes. In accordance with the published study protocol, we examined whether sociodemographic and clinical factors–including age, sex, relationship status, educational level, ECOG PS, tumour subtype, number of metastatic sites, radiological response–were associated with baseline EORTC QLQ-C30 GHS and other function and symptom scores, as well as their change over time. LDH >250 U/L, lymphocytopenia and hypoalbuminaemia were excluded due to the substantial amount of missing data and the few patients meeting the threshold.

In order to examine differences in the trend of scores over time between groups we considered the interaction of clinical and sociodemographic factors with time. For each variable, we first fitted a univariate model without interaction. We then added a time interaction term and used ANOVA to compare model fit. The model with the better fit was used for interpretation and visualisation. A significant time interaction indicated a difference in the rate of change over time between the reference group and another subgroup. For variables with more than two levels (e.g. sarcoma subtype), we redefined the reference level to explore all relevant comparisons. If the model without interaction was retained, this indicated a similar change over time. For the multivariable model selection we used the backward selection method (p < 0.05).

The following post-hoc exploratory analyses were conducted. First, country of recruitment (UK vs NL) was added as a fixed effect and as an interaction with time in the LME models to explore potential confounding by recruitment country. Second, pain scores were examined according to radiological response.

GH and pain scores will be reported in detail in the main text. The other domains will be reported more extensively in the Supplementary Appendix. Statistical analyses were performed using R v.4.4.2 (R Development Core Team and the R Foundation for Statistical Computing) by integration of software from open-source packages, including nlme, and packages from tidyverse, including dplyr, tidyr and ggplot2.

### Role of the funding source

The funders were given the opportunity to review the manuscript, but did not influence the study design, data collection, analysis, or interpretation.

## Results

### Sociodemographic and clinical characteristics

**Fig. 1:**
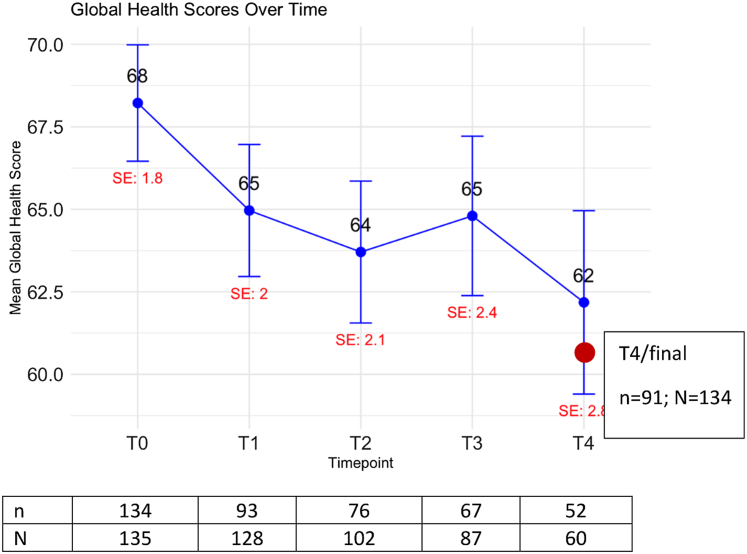
**Global Health Scores (GHS) over time**. Mean GHS and standard errors (SE) for the 5 time points (T0, T1, T2, T3, T4) are represented on the y-axis. For patients who did not complete 4 cycles, the last score post baseline was used (i.e. T4/final). Mean GHS for T4/final is 60.7 (SE 2.1). The mean difference between T0 and T4/final is −11.3 points (p < 0.001). n represents the number of patients who completed the questionnaire at baseline (T0), cycle 1 (T1), 2 (T2), 3 (T3) and 4 (T4), respectively. N represents the number of patients who were still on treatment at that time point. For T4/final, 91 observed measurements were available out of 134 patients with at least one post-baseline measurement (68%).

The HOLISTIC study included 137 patients of the UK (n = 72) and the Netherlands (n = 65). Two patients never started chemotherapy and were excluded. Forty-four percent (60/135) of patients had at least 4 cycles of chemotherapy. Questionnaire completion rates decreased over time, with 134 patients completing the baseline questionnaire (T0), 93 at T1, 76 at T2, 67 at T3, and 52 at T4. The number of patients eligible to complete the questionnaire (i.e. still on treatment at that time point) is indicated in Fig. 1.

91 (68%) of 134 patients filled out the questionnaire at T0 together with at least the questionnaire at T2, T3 and/or T4 (i.e. T4/final). In 35/135 patients the questionnaire was only filled out at T0 and one patient only filled out the questionnaire at T1. For these patients, study drop out was attributed to death (n = 12), best supportive care (n = 6), the patient being too unwell to continue the study (n = 2), patient preference (n = 4), or the reason was missing (n = 12).

Sociodemographic and clinical characteristics of included patients are shown in Table 1. Most patients were aged between 40 and 65 (51.1%) or > 65 (40.7%) years and sex distribution was even. In the eldest group (>65 years) more patients had an ECOG PS 1/2 as compared to patients ≤65 years (65.5% and 55.0%, respectively). Six patients had an ECOG PS of 2. The most common STS subtypes were leiomyosarcoma (LMS, 29.6%), liposarcoma (LPS, 22.2%), and undifferentiated pleomorphic sarcoma (UPS, 12.6%). In 57% of cases the interval between diagnosis of advanced STS and study participation was >6 months. 64% (86/135) of patients were treated with monotherapy, with doxorubicin being used in 80% of these cases. Patients with ECOG PS 0 or with ECOG PS 1 or 2 were treated with combination regimens in 37% (16/43) and in 36% (29/80) of cases, respectively (Supplementary S1).

**Table 1:** Patient demographic and clinical characteristics.

	N (%)
**Country**	
United Kingdom	72 (53.3%)
Netherlands	63 (46.7%)
**Sex**	
Male	67 (49.6%)
Female	68 (50.3%)
**Educational level**	
Low	27 (20.0%)
Medium	77 (57.0%)
High	31 (23.0%)
**Relationship status**	
Partner	112 (83.0%)
No partner	23 (17.0%)
**Age**	
18–39	11 (8.1%)
40–65	69 (51.1%)
>65	55 (40.7%)
**ECOG performance status**	
0	43 (31.9%)
1	74 (54.8%)
2	6 (4.4%)
Missing	12 (8.9%)
**Number of metastatic sites**	
≤2	108 (80.0%)
>2	27 (20.0%)
**Anaemia**^a^	
No	88 (65.2%)
Yes	44 (32.6%)
Missing	3 (2.2%)
**Lymphopenia**^b^	
No	85 (63.0%)
Yes	17 (12.6%)
Missing	33 (24.4%)
**LDH >250 U/L**^c^	
No	96 (71.1%)
Yes	16 (11.9%)
Missing	23 (17.0%)
**Hypoalbuminaemia**^d^	
No	108 (80%)
Yes	13 (9.6%)
Missing	14 (10.4%)
**Histological subtype**	
Leiomyosarcoma	40 (29.6%)
Liposarcoma	30 (22.2%)
UPS^e^	17 (12.6%)
Angiosarcoma	8 (5.9%)
Other^f^	40 (29.6%)
**Treatment regimens**	
*Monotherapy*	**86 (100)**
Liposomal doxorubicin	1 (1)
Doxorubicin	69 (80)
Eribulin	1 (1)
Ifosfamide	3 (3)
Paclitaxel	9 (10)
Gemcitabine	1 (1)
Trabectedin	1 (1)
Sirolimus	1 (1)
*Combination therapy*	**49 (100)**
Doxorubicin-ifosfamide	9 (18)
Gemcitabin-docetaxel	1 (2)
Olaratumab-doxorubicin	30 (61)
Doxorubicin-dacarbazine	6 (12)
Gemcitabin-pembrolizumab	2 (4)
Gemcitabin-dacarbazine	1 (2)
**Response**^g^	
Partial response	21 (15.6%)
Stable disease	46 (34.1%)
Progressive disease	49 (36.3%)
Missing	19 (14.1%)

a Anaemia: Haemoglobin <13 g/L male, <11.5 g/L female).

b Lymphopenia <1 × 10^9^/L.

c LDH = lactate dehydrogenase.

d Hypoalbuminaemia <35 g/L.

e Undifferentiated pleomorphic sarcoma.

f Other histological subtypes include: clear cell sarcoma, endometrial stromal sarcoma, epithelioid haemangioendothelioma, fibrosarcoma, Kaposi sarcoma, malignant peripheral nerve sheath tumour, myxofibrosarcoma, solitary fibrous tumour, synovial sarcoma, sarcoma not otherwise specified.

g Radiological response was defined by RECIST (version 1.1) criteria.

No significant differences were observed between patients in the UK and the Netherlands in terms of age, sarcoma subtype distribution, ECOG PS, disease stage or treatment regimens.^13^ Also, there were no significant differences for anaemia or hypoalbuminaemia at baseline (anaemia: UK 23/70 = 32.9% vs NL 21/61 = 33.9%, p = 1; hypoalbuminaemia: UK 8/70 = 11.4% vs NL 5/51 = 9.8%, p = 1).

In 19/135 patients, the first response evaluation was missing. In 42.2% of patients (49/116) the first response evaluation indicated PD whereas in 18.1% and 39.7% of patients PR or SD was observed, respectively. In 73 out of 135 patients (54.1%) first response evaluation after starting first-line palliative chemotherapy was available together with the result of the questionnaire. In 43 patients first response evaluation was available but no questionnaire was completed at this time point. The questionnaire was missing in 20/49 (40.8%) patients with PD and in 23/67 (34.3%) patients with PR or SD.

At T4, in most cases (64.4%, 47/73 patients), the response evaluation was close to this time point. In the remaining patients the questionnaire closest to response evaluation was the questionnaire filled out at T2 (5/74), T3 (18/74) or 3 weeks after the end of chemotherapy (3/74).

### Primary endpoint: Global Health descriptive statistics

Mean GHS for T0-T4 are shown in Fig. 1. The mean GHS statistically and clinically worsened over time, with a mean score of 68.2 at T0 and 60.7 at T4/final, indicating a mean difference of −11.3 points between T0 and T4/final (95% CI 7.6–15.0, t-test p < 0.001). A sensitivity analysis including only patients for which T4 was available showed a mean difference between GH at T0 and T4 of 9.8 points (p < 0.001). Both in the group of patients with PR or SD (n = 44, mean difference −12.3 points, 95% CI 7.9–16.8, p > 0.001) and in the group with PD (n = 29, mean difference −10.9, 95% CI 2.3–19.6, p = 0.015) a statistically and clinically significant worsening of GHS was observed (Supplementary S2).

### Secondary endpoints

#### Descriptive statistics for EORTC QLQ-C30 symptom and function domains

For fatigue, nausea and vomiting, dyspnoea and constipation similar trends were seen as for GH (Supplementary S3). For pain scores a statistically significant (p = 0.049) but not clinically meaningful improvement over time was seen. For insomnia and diarrhoea, no significant changes over time were observed. For appetite loss and most function scores only statistically (but not clinically relevant) significant worsening over time was reported (Supplementary S4).

#### Global Health: univariate and multivariable analysis (linear mixed effect models)

**Fig. 2:**
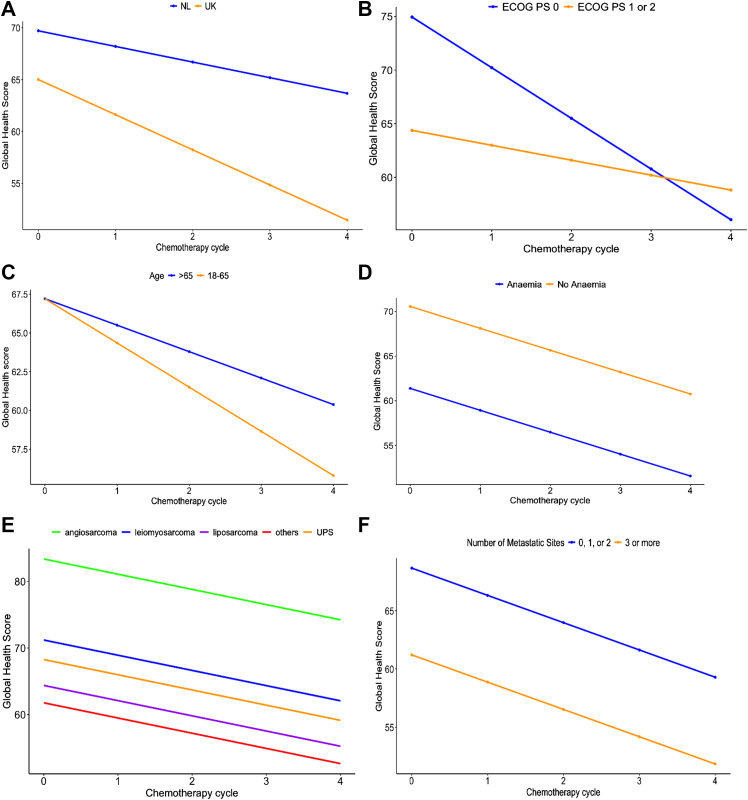
**Global Health Scores (GHS) over time, using linear mixed effect model**. **A)** No significant baseline difference in GHS between the UK and NL (p = 0.159) and no difference in the change rate over time between subgroups (p = 0.1152). **B)** Patients with ECOG PS 0 have higher baseline GHS than those with ECOG PS 1–2 (p = 0.002). GHS decrease over time for ECOG PS 0 but remain unchanged for ECOG PS 1–2 (p = 0.0139). **C)** No significant difference at baseline or over time between age groups (p > 0.05). **D)** Patients with anaemia at baseline have lower baseline GHS (p = 0.004) but there is no significant difference in the decrease rate of GHS. **E)** Patients with angiosarcoma have significantly higher baseline GHS than most subgroups (p < 0.05), except leiomyosarcoma (p = 0.057); no change over time (p > 0.05). **F)** Patients with <3 metastatic sites tend to have higher baseline GHS (p = 0.052), with no significant difference in change over time (p = 0.882). ECOG PS = Eastern Cooperative Oncology Group ECOG Performance Status; LME = Linear mixed effect model; UK = United Kingdom; NL = the Netherlands.

According to univariate analysis, baseline GHS were significantly lower (i.e. worse) for patients with ECOG PS 1 or 2 (Fig. 2B) and anaemia (Fig. 2D). At baseline, GHS for patients with angiosarcoma were significantly higher compared to all other sarcoma subgroups except for LMS (p = 0.057) (Fig. 2E). Patients with >2 metastatic sites showed some evidence (p = 0.052) of an association with lower baseline GHS (Fig. 2F). In the univariate analysis there was no significant difference in baseline GHS between patients in the UK and the Netherlands (p = 0.159) (Fig. 2A). For relationship status and educational level there were no significant differences between subgroups at baseline (relationship status: p = 0.901; educational level: p = 0.504) or over time (relationship status: p = 0.485; educational level: p = 0.401). GHS decreased over time (p < 0.001). Interaction analysis showed a faster worsening of GHS in patients with ECOG PS 0 compared to those with ECOG PS 1–2 (p = 0.014, Fig. 2B). For the remaining variables there were no significant differences in GH scores at baseline or change rates over time. GHS also worsened over time in both patients with PR or SD and in those with PD (p < 0.05), with no significant difference between the groups in either baseline scores or change rate over time (not shown in figure).

According to multivariable analysis baseline GHS were worse for patients from the UK, patients with number of metastatic sites >2, ECOG PS 1 or 2, anaemia, or the tumour subgroup ‘other’ (Table 2). Patients with LPS showed a tendency towards lower baseline GHS (p = 0.063). Generally, GHS decreased over time. GHS decreased faster (p < 0.05) in patients with ECOG PS 0 compared to patients with ECOG PS 1 or 2.

**Table 2:** Multivariable analysis of Global Health Scores (GHS) with Linear Mixed Effect Model (LME).

Multivariable analysis of Global Health scores (GHS)	Estimate^a^	Confidence interval	P-value
**Intercept**^b^ (NL, ECOG PS 0, LMS, = ≤2 metastatic sites, no anaemia)	84.7	77.1–92.2	**<0.001**
**Time** (per cycle, reference group)	−4.7	−6.9 to −2.6	**<0.001**
**Country** (ref = NL)	−7.3	−12.7 to −2.0	**0.008**
**Number of metastatic sites** (ref = ≤2)	−8.5	−15.5 to −1.5	**0.019**
**Anaemia** (ref = no anaemia)	−6.1	−12.0 to −0.3	**0.040**
**ECOG PS** (ref = ECOG PS 0)	−7.6	−14.0 to −1.1	**0.023**
**Histological subtype** (ref = LMS)			
Angiosarcoma	+7.5	−4.3 to −19.4	0.218
Liposarcoma	−7.3	−14.9 to −0.3	0.063
UPS	−2.7	−11.7 to 6.4	0.566
Other	−7.8	−14.9 to −0.7	**0.035**
**Time∗ECOG PS** (ref = ECOG PS 0)	3.3	0.7–6.0	**0.014**

ECOG PS = Eastern Cooperative Oncology Group ECOG Performance Status; LME = Linear mixed effect model; LMS = Leiomyosarcoma; UPS = Undifferentiated pleomorphic sarcoma.

Worse baseline GHS for patients with number of metastatic sites >2 (76.2 vs 84.7), anaemia (78.6 vs 84.7), ECOG PS 1 or 2 (77.1 vs 84.7), or the tumour type subgroup ‘other’ (76.9 vs 84.7), and for UK vs NL (77.4 vs 84.7). Over time, GHS decreased in the reference group (NL, ECOG PS 0, LMS, = ≤2 metastatic sites, no anaemia) at a rate of −4.7 points per cycle. Faster decrease of GHS in patients with ECOG PS 0 (change rate −4.7) compared to patients with ECOG PS 1 or 2 (change rate −1.4).

Bolded P values indicate statistical significance (P < 0.05).

a Relatively to reference level.

b Corresponding to the reference.

#### Symptom domains: univariate analysis (linear mixed effect models)

**Fig. 3:**
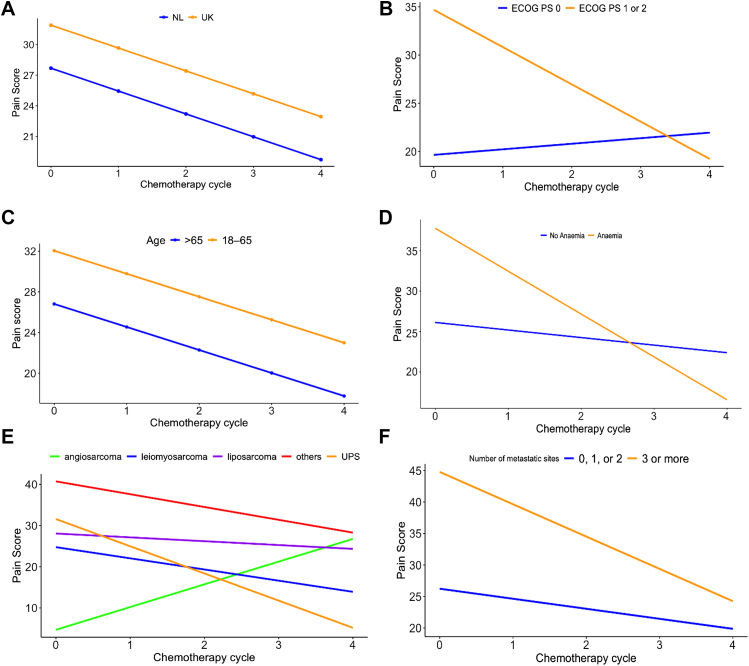
**Pain scores over time, using linear mixed effect models**. **A)** No difference in baseline pain scores (p = 0.311) or in the change rate over time between patients in the UK and Netherlands (0.450). **B)** Higher baseline pain scores in patients with ECOG PS 1–2 (34.7 vs 19.7 [ECOG PS 0], p = 0.005). Decrease over time of pain scores in patients with ECOG PS 1–2 compared to patients with ECOG PS 0 (p = 0.013). **C)** No difference in baseline pain scores (p = 0.211) or in the change rate over time between age groups (0.625). **D)** Higher baseline pain scores for patients with anaemia (37.8 vs 26.2 [anaemia] p = 0.031). Decrease over time of pain scores in anaemic compared to non-anaemic patients (p = 0.012). **E)** Lower baseline pain scores for angiosarcoma (4.7) patients compared to liposarcoma (28.1, p = 0.042), UPS (31.6, p = 0.030), and ‘other’ sarcoma subtypes (40.7, p = 0.001). Patients with ‘other’ sarcomas had higher baseline pain scores than LMS patients (24.7, p = 0.014). Decrease of pain scores over time in UPS (p = 0.004) and ‘other’ sarcomas (p = 0.024). Stable pain scores for LMS (p = 0.066), LPS (p = 0.575), and angiosarcoma (p = 0.054). **F)** Higher baseline pain scores in patients with >2 metastatic sites (44.7 vs 26.2 [>2 sites], (p = 0.031). No significant difference in the change rate over time (p = 0.089). ECOG PS = Eastern Cooperative Oncology Group ECOG Performance Status; LME = Linear mixed effect model; UK = United Kingdom; NL = The Netherlands; UPS = Undifferentiated pleomorphic sarcoma; LMS = leiomyosarcoma.

Baseline pain scores were worse in patients with >2 metastatic sites (44.7 vs 26.2 [≤2 sites], (p = 0.031), anaemia (37.8 vs 26.2 p = 0.031) or ECOG PS 1–2 (34.7 vs 19.7 [ECOG PS 0], p = 0.005), but lower in patients with angiosarcoma (4.7 vs 28.1, p = 0.042 [LPS], 31.6, p = 0.030 [UPS], 40.7, p = 0.001 [other]) (Fig. 3). Generally, pain scores improved over time (p = 0.005). Pain scores improved faster for patients with ECOG PS 1–2 (p = 0.013), patients with anaemia (p = 0.031), UPS (0.004) or ‘other’ sarcoma (p = 0.024). Pain scores improved both in patients with PR/SD and patients with PD, without a difference in the change rate over time between subgroups (p = 0.278).

Worse baseline fatigue scores were shown in UK patients (41.7 vs 31.6, p-value = 0.010), those with anaemia (46.1 vs 32.0, p = 0.002), aged 18–65 years (40.4 vs 32.0, p = 0.035) and patients with ECOG PS 1–2 (40.8 vs 28.8 [ECOG PS 0], p = 0.012) (Supplementary S5). Patients with angiosarcoma had lower baseline fatigue scores than all other subgroups (p < 0.05). Generally, fatigue scores worsened over time (p < 0.05). Fatigue scores worsened faster in patients with ECOG PS 0 (change rate 8.3 vs 2.4 [ECOG PS 1–2], p < 0.001), and in non-anaemic patients compared to anaemic patients (change rate 5.5 vs 1.9, p = 0.029). For patients with UPS there was no change in fatigue scores over time (change rate −0.4, p = 0.874).

Patients with ECOG PS 1 or 2 and UPS had lower baseline dyspnoea scores. Generally, dyspnoea scores did not change over time (p < 0.05), with worsening observed only in some subgroups including patients aged 18–65 years, those with ECOG PS 0, normal baseline haemoglobin, patients with angiosarcoma or LPS (Supplementary S6).

Baseline appetite scores were similar across groups (Supplementary S7). Appetite loss scores remained stable over time, except for a worsening in patients with ECOG PS 0 or without baseline anaemia. Generally, there was a worsening of constipation (Supplementary S8) and nausea and vomiting (Supplementary S9) scores over time. Insomnia scores remained stable over time for nearly all subgroups (Supplementary S10).

#### Function domains: univariate analysis (linear mixed effect models)

Baseline physical function scores were worse for female patients (75.3 vs 82.5, p = 0.036), those with ECOG PS 1 or 2 (86.5 vs 74.3, p = 0.002) or those with the STS subtype ‘other’ (73.9 vs 89.4, p = 0.0427 [angiosarcoma]; 84.2, p = 0.022 [LMS] (Supplementary S11). Physical function scores worsened over time with a faster decline in patients with ECOG PS 0 compared to those with ECOG PS 1 or 2 (change rate −3.4 vs −2.2, p = 0.022). Social function scores did not change over time (p > 0.05) except for patients with ECOG PS 0 (i.e. worsening), anaemia (i.e. improvement), no anaemia (worsening) (Supplementary S12). Role function scores worsened over time, except for patients with a high educational level (i.e. stable) (Supplementary S13). A sharper decline was observed in patients with ECOG PS 0 compared to those with ECOG PS 1–2. Emotional function scores were stable for most subgroups (Supplementary S14), whereas cognitive function scores worsened over time (Supplementary S15).

## Discussion

Here we present for the first time the course of GHS in a multicentre international study in patients with advanced STS treated with the standard first-line chemotherapy. Overall, patients with STS receiving first-line palliative chemotherapy experience a decrease in GHS over time. Fitter patients (i.e. ECOG PS 0) are more likely to experience a faster decline in GHS. This finding is crucial for the decision-making process, especially considering the limited OS benefit provided by first-line palliative chemotherapy in this population and chemotherapy toxicity. Moreover, the results suggest that postponing therapy could be a viable option for certain patients, given the fact that the GHS of patients with ECOG PS 1-2 decreases to a lesser extent, allowing them more time to consider their treatment options and avoid the side effects of chemotherapy for a period.

Multiple reasons might explain the more rapid deterioration in GHS and physical function scores among fitter patients. While fitter patients may receive higher chemotherapy doses or more often combination treatment, combination therapy rates were similar across ECOG groups. Patients with ECOG PS 0 start with better baseline GHS, and may, therefore, perceive the worsening of GH and symptoms as more pronounced compared to patients with ECOG PS 1 or 2. Interestingly, patients with ECOG PS 0 at baseline have similar or even higher GHS scores than the general population in the UK (mean GHS 62.3) and in the Netherlands (mean GHS 77.4).^21^ Additionally, fitter patients might be more active initially. Hence, chemotherapy toxicity might have a greater impact on their GHS and physical function scores. Similar findings were reported in another study, where chemotherapy did not improve QoL in patients with ECOG PS 2 and worsened near death in patients with ECOG PS 1, underscoring the negative consequences of continuing chemotherapy near the end of life.^22^

GHS decrease over time in patients with PD as well as those with PR or SD, suggesting that chemotherapy toxicity contributes to this deterioration. However, in a real-word setting, the decrease in GHS might be more pronounced in patients with PD, though this effect may not have been fully captured due to missing data. These findings suggest that RECIST may not be an adequate surrogate to assess the clinical benefit of chemotherapy, as it does not capture symptom burden or treatment-related toxicity. For instance, a recent study of MEK inhibition in patients with epithelioid hemangioendothelioma demonstrated limited RECIST responses, yet patients reported a significant reduction in pain intensity.^23^ This underscores that clinical benefit cannot be determined by RECIST (or survival outcomes) alone and highlights the importance of systematically integrating PROs into clinical trials and routine care.

As longitudinal QoL data in patients with STS on systemic therapy are lacking, we compared our results with studies evaluating QoL in patients with other cancer types. A study in colorectal cancer patients receiving palliative chemotherapy showed improved QoL in patients with a treatment response as compared to patients with PD.^24^ Similarly, in another study, QoL worsened over time in patients receiving best supportive care, as expected with disease progression.^25^ However, data comparing QoL decline in patients with STS on BSC vs chemotherapy are lacking. Moreover, direct comparisons with the HOLISTIC study are challenging, as patients with sarcoma may experience symptoms that impact QoL differently from those with more common tumour types.

Surprisingly, there was no significant difference in the change rate of GHS over time between patients aged 18–65 years and those >65 years. This suggests older age alone may not be a barrier to consider palliative chemotherapy. However, these results should be interpreted cautiously, as less fit elderly patients likely weren't included, and chemotherapy dose data were missing. Still, in our cohort, elderly (>65 years) and younger (18–65 years) patients equally often received combination therapy. Moreover, elderly patients had similar baseline GHS, compared to younger patients. Nevertheless, the majority of patients >65 years had ECOG PS 1 or 2 (36/55).

In the multivariate model, at baseline, GHS were significantly worse in UK patients compared to NL patients. The reasons for this difference may be diverse and not only cancer specific. It has been described that the GHS in the general population is much lower in the UK than in the Netherlands.^21^ For metastatic STS there is no real world data available from other data sets in the first line palliative systemic treatment setting and also for other tumour types data in this context are lacking. An explanation maybe that 65% of UK patients had been diagnosed more than six months before participating in the HOLISTIC study, compared to 48% in the Netherlands (p = 0.041), indicating that UK patients were further along in their disease trajectory at study entry and potentially experiencing a higher symptom burden at baseline. In an earlier publication of the HOLISTIC baseline data, no significant differences were found between UK and Dutch patients in terms of age, sarcoma subtype, or ECOG PS.^13^ Differences in comorbidity and socio-economic status between patients from both countries could have played a role, but this is all purely speculative as data from the HOLISTIC study population on these relevant topics is not available.

While overall pain scores improved over time, this improvement may be attributed to factors such as pain medication, palliative radiotherapy, and the presence of the primary tumour, variables that were not registered in this study. Patients with more advanced disease (i.e.> 2 metastatic sites, ECOG PS 1 or 2) experienced a faster decline in pain scores, possibly due to earlier and more effective pain management. Pain relief should be considered during the decision-making process about palliative chemotherapy, but future research is needed to explore the impact of chemotherapy on pain, considering pain management strategies and tumour response.

Earlier studies showed contrasting results regarding the role of PROs in treatment decisions for patients receiving palliative chemotherapy.^26^, ^27^, ^28^ Although most physicians acknowledge the use of PROs, they rarely influence the decision-making process. A Dutch study, found that 70% of patients receiving palliative chemotherapy, despite seriously impaired HRQoL, but without evidence of tumour progression or toxicity, continued treatment as planned.^26^ In contrast, tumour progression and serious treatment toxicity almost always influence treatment decisions. Given the increasing implementation of PROs in clinical practice, future research should not only focus on collecting and analysing PROs, but also on how to effectively incorporate PRO data into the shared decision-making process.

Almost one third of patients had anaemia at baseline (i.e. before starting palliative chemotherapy), aligning with the presence of advanced disease. These patients also had worse baseline GHS, possibly due to anaemia contributing to symptoms like fatigue and dyspnoea.^29^^,^^30^ Fatigue and dyspnoea scores worsened faster in patients without anaemia, possibly due to chemotherapy-induced anaemia. Interventions like transfusions or erythropoietin may improve HRQoL, but the lack of transfusion data limits the ability to interpret the effect of haemoglobin levels on HRQoL.

Overall, GHS and most symptom scores, including nausea, vomiting, fatigue, dyspnoea, and constipation, worsened over time. Proactive side-effect management may help improve HRQoL. In contrast, most function scores remained stable. This could be seen as a positive outcome, indicating that functional scores remain relatively stable throughout palliative chemotherapy.

Several factors might have contributed to an underestimation of the decline in GHS. First, one-fourth of patients completed only the baseline questionnaire, often due to deterioration or death, meaning those in worse condition were more likely to leave the study. Since the LME model assumes data are missing at random, this may bias results.

A limitation of this study was that we did not register how many patients declined to participate or their reasons for doing so. This may have introduced selection bias, as patients with more severe symptoms or lower HRQoL may have been less inclined to participate. In addition to this, a relatively high dropout rate–primarily due to disease progression, clinical deterioration, or death–further limited the dataset. To address this, PROs should be integrated into routine palliative care, as recommended by the European Association for Palliative Care (EAPC), using validated questionnaires focusing on symptoms relevant for patients in a palliative care setting, using flexible administration methods (e.g. paper, online, telephone), while limiting patient burden.^31^ Furthermore, electronic PROs (ePROS) have demonstrated their utility in routine clinical care in the advanced and metastatic setting.^32^

Another limitation was the small sample size, though participation rates are comparable to those reported in other studies involving patients with advanced cancers.^25^ A larger study, including patients from both European and non-European countries, would enable more powerful subgroup analyses and help identify patients that benefit from palliative chemotherapy. It would have been valuable to examine GHS change after the end of chemotherapy, particularly in patients with PR or SD as this could explain to which extent the deterioration of GHS is caused by chemotherapy toxicity. However, most patients dropped out after cycle 4 or earlier. Furthermore, we did not report chemotherapy dose reductions, which might also impact GHS. Future research should investigate if the (change in) HRQoL influences treatment decisions. Also, it remains challenging to determine the perfect timing of QoL assessment, as our cycle-based approach may have missed interim changes.

Despite these limitations, we have shown for the first time what the impact is of standard first-line palliative chemotherapy in routine clinical practice on patients with STS HRQoL, which adds much to the shared discussion with the patients whether or not to start (anthracycline containing) chemotherapy in this setting.

In conclusion, first-line palliative systemic treatment in advanced STS is associated with a significant decrease in GHS. Fitter patients (i.e. ECOG PS 0) are more likely to experience a faster decline in GHS, symptom and function scores. GHS decrease over time in patients with PD as well as in those with PR or SD, indicating that chemotherapy toxicity contributes to the decline in GH. These results emphasise the importance of integrating PROs in clinical trials and routine care. When counselling patients on the potential risks and benefits of palliative chemotherapy, clinicians should communicate the possibility of a decline in QoL due to chemotherapy.

## Contributors

All authors listed have provided substantial contributions to this manuscript. ER contributed to the methodology, curated the data, performed formal analyses, and wrote the original draft of the manuscript. EY contributed to the conceptualisation of the study, recruited patients, curated data, and reviewed and edited the manuscript. RLJ contributed to data curation and reviewed and edited the manuscript. DH contributed to patient recruitment and data curation. LA performed formal analyses, created visualisations, and reviewed and edited the manuscript. IMED contributed to patient recruitment and reviewed and edited the manuscript. RJY contributed to patient recruitment and reviewed and edited the manuscript. AWO contributed to patient recruitment and reviewed and edited the manuscript. JJH contributed to patient recruitment and reviewed and edited the manuscript. HG contributed to patient recruitment and reviewed and edited the manuscript. NS reviewed and edited the manuscript. OH contributed to conceptualisation, methodology, supervised the study, and reviewed and edited the manuscript. WTA contributed to conceptualisation, methodology, supervised the study, and reviewed and edited the manuscript. EY and ER accessed and verified the underlying data. All authors have read and approved the final version of the manuscript.

## Data sharing statement

The study protocol has been published. Deidentified participant data and the statistical analysis plan are available upon reasonable request. Requests should be directed to the corresponding author at w.t.a.vandergraaf@erasmusmc.nl and will be considered following approval of a proposal. Data access will require a signed data access agreement.

## Declaration of interests

N Steeghs provided consultation or attended advisory boards for Bayer, Boehringer Ingelheim, Bristol-Myers Squibb, Ellipses Pharma, GlaxoSmithKline, Incyte. NS received research grants from Abbvie, Actuate Therapeutics, Amgen, Anaveon, AstraZeneca, Bayer, Blueprint Medicines, Boehringer Ingelheim, Bristol-Myers Squibb, CellCentric, Cogent Biosciences, Cresecendo Biologics, Daiichi Sayko, Deciphera, Exelixis, Genentech, GlaxoSmithKline, Iambic, IDRx, Immunocore, Incyte, Janssen, Kling Biotherapeutics, Lixte, Merck, Merck Sharp & Dohme, Merus, Molecular Partners, Novartis, Pfizer, Revolution Medicin, Roche, Sanofi, Zentalis. All outside the submitted work, all payment to the Netherlands Cancer Institute. All outside the submitted work, all payment to the Netherlands Cancer Institute. WTA: research grants from Eli Lilly (to the Institute). All remaining authors have declared no conflicts of interest.
