# Supplementary material for: Health-related quality of life in patients with advanced soft tissue sarcoma receiving first-line palliative chemotherapy (HOLISTIC): longitudinal results from a prospective, observational cohort study
Source: eClinicalMedicine. 2025 Oct 16;89:103561. doi: 10.1016/j.eclinm.2025.103561 (PMC12554115; doi:10.1016/j.eclinm.2025.103561)
Supplement: Supplementary [file mmc1.docx]

# Supplementary

## Supplementary 1 : treatment regimens according to ECOG PS

| Chemotherapy regimen according to ECOG PS | | | |
| --- | --- | --- | --- |
|  | **Monotherapy**, n (%) | **Combination therapy**, n (%) | **Total, n (%)** |
| **ECOG PS 0** | 27 (63%) | 16 (37%) | 43 (100%) |
| **ECOG PS 1 or 2** | 51 (64%) | 29 (36%) | 80 (100%) |

## Supplementary 2 Global Health Scores (GHS) in patients with Partial Response (PR) or Stable Disease (SD) versus patients with Progressive Disease (PD).

|  | **GHS according to radiological response** | | | | | | | |
| --- | --- | --- | --- | --- | --- | --- | --- | --- |
|  | **Patients with PR or SD (n=44)** | | | | **Patients with PD (n=29)** | | | |
| **Time point** | **Mean** | **SE** | **Mean difference T0 vs T_response** | **P-value*** | **Mean** | **SD** | **Mean difference T0 vs T_response** | **P-value*** |
| T0 | 73.7 | 2.3 | **-12.3** | **<0.001** | 70.7 | 3.6 | **-10.9** | **0.015** |
| Response evaluation* | 61.4 | 2.6 |  |  | 59.8 | 4.4 |  |  |

*Time point closest to response evaluation (in most cases T4).

SE=standard error

## Supplementary 3 : Descriptive statistics EORTC QLQ-C30 symptom domains


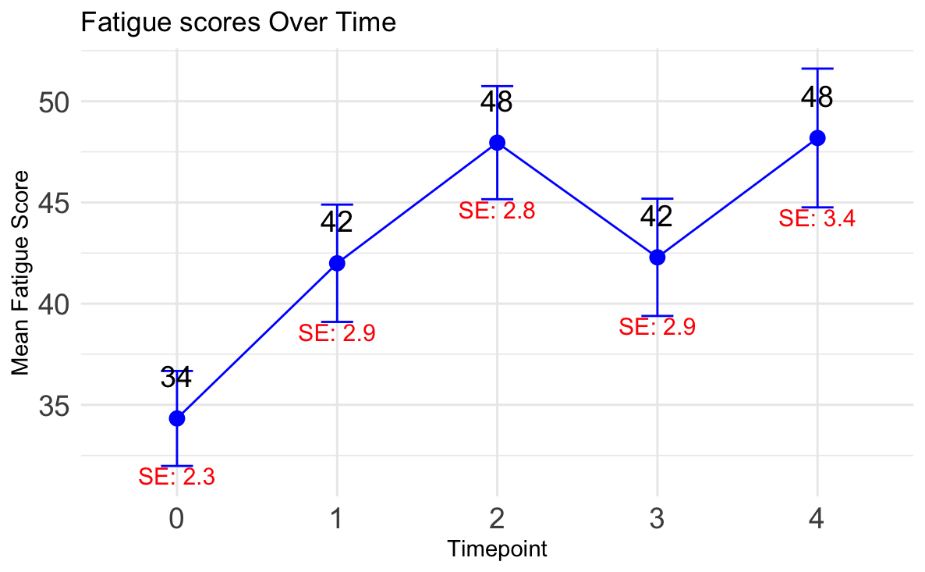


SE = standard errror

Fatigue scores over time. Mean scores for the 4 time points (T0, T1, T2, T3, T4) are represented on the y- axis. Mean score for T4 artificial is 49.8 (SE 2.6). The mean difference between T0 and T4 artificial is +19.2 points (p<0.001).


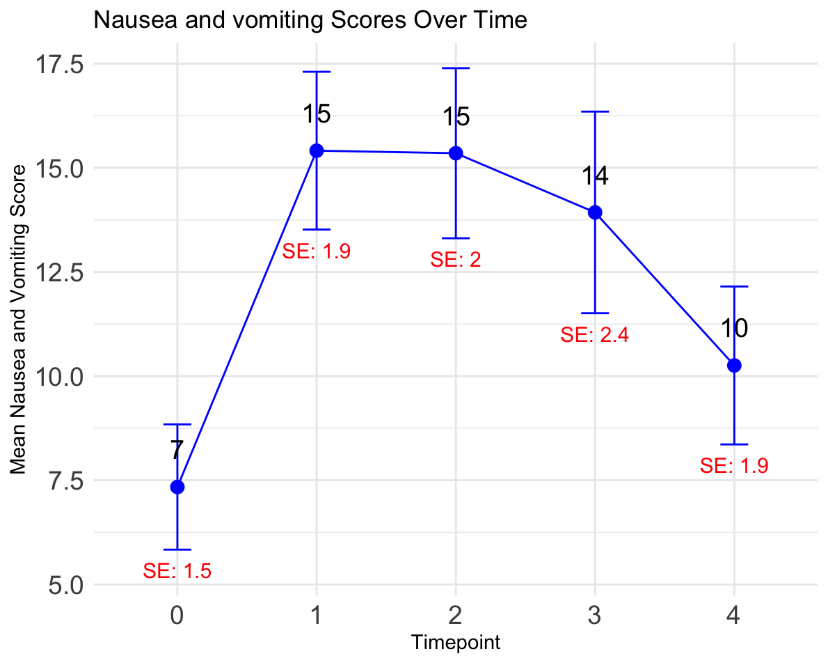


SE = standard errror

Nausea and vomiting scores over time. Mean scores for the 4 time points (T0, T1, T2, T3, T4) are represented on the y- axis. Mean score for T4 artificial is 13.9 (SE 2.06). The mean difference between T0 and T4 artificial is +9.1 points (p<0.001).


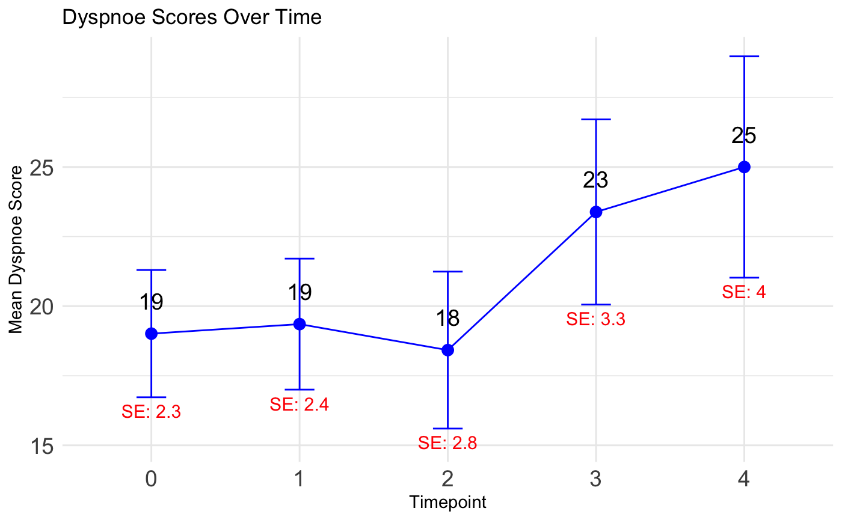


SE = standard errror

Dyspnoe scores over time. Mean scores and standard error for the 4 time points (T0, T1, T2, T3, T4) are represented on the y- axis. Mean score for T4 artificial is 24.5 (SE 2.9). The mean difference between T0 and T4 artificial is +10.3 points (p=0.001).


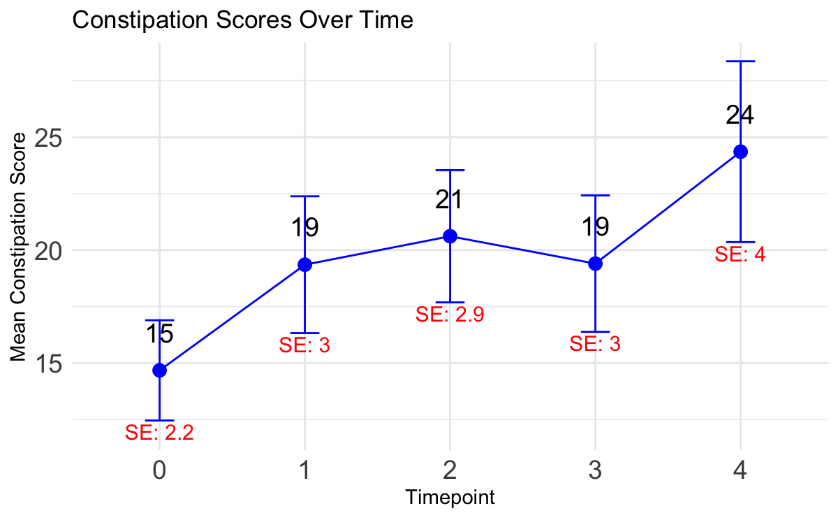


SE = standard errror

Constipation scores over time. Mean scores for the 4 time points (T0, T1, T2, T3, T4) are represented on the y- axis. Mean score for T4 artificial is +21.8 (SE 2.49). The mean difference between T0 and T4 artificial is +13.3 points (p**<0.001**).


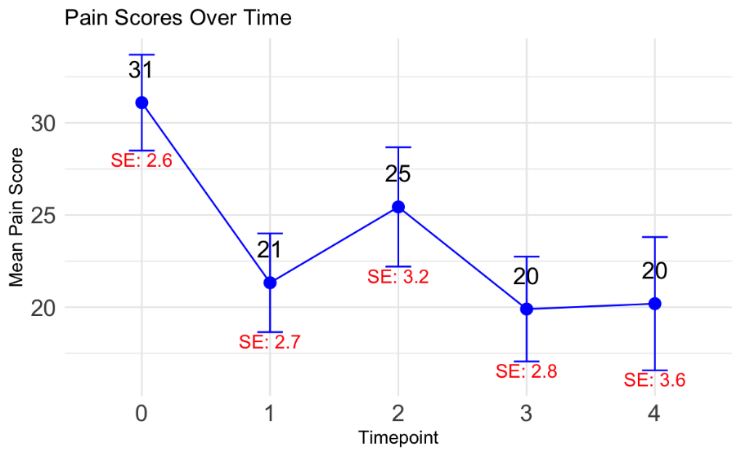


SE = standard errror

Pain scores over time. Mean pain scores and standard error for the 4 time points (T0, T1, T2, T3, T4) are represented on the y- axis. Mean GH score for T4 artificial is 21.8 (SE 2.49). The mean difference between T0 and T4 artificial is -5.9 points (p=0.04919).


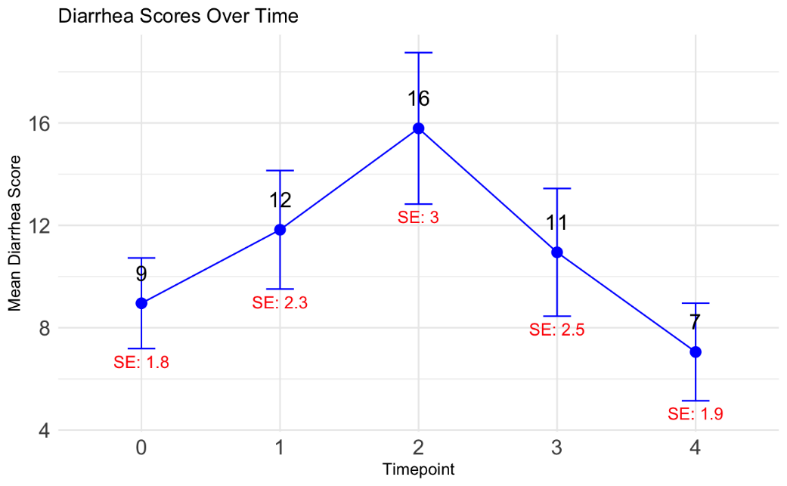


SE = standard errror

Diarrhea scores over time. Mean scores for the 4 time points (T0, T1, T2, T3, T4) are represented on the y- axis. Mean score for T4 artificial is 9.9 (SD 19.6). The mean difference between T0 and T4 artificial is -3.3 points (p=0.186).


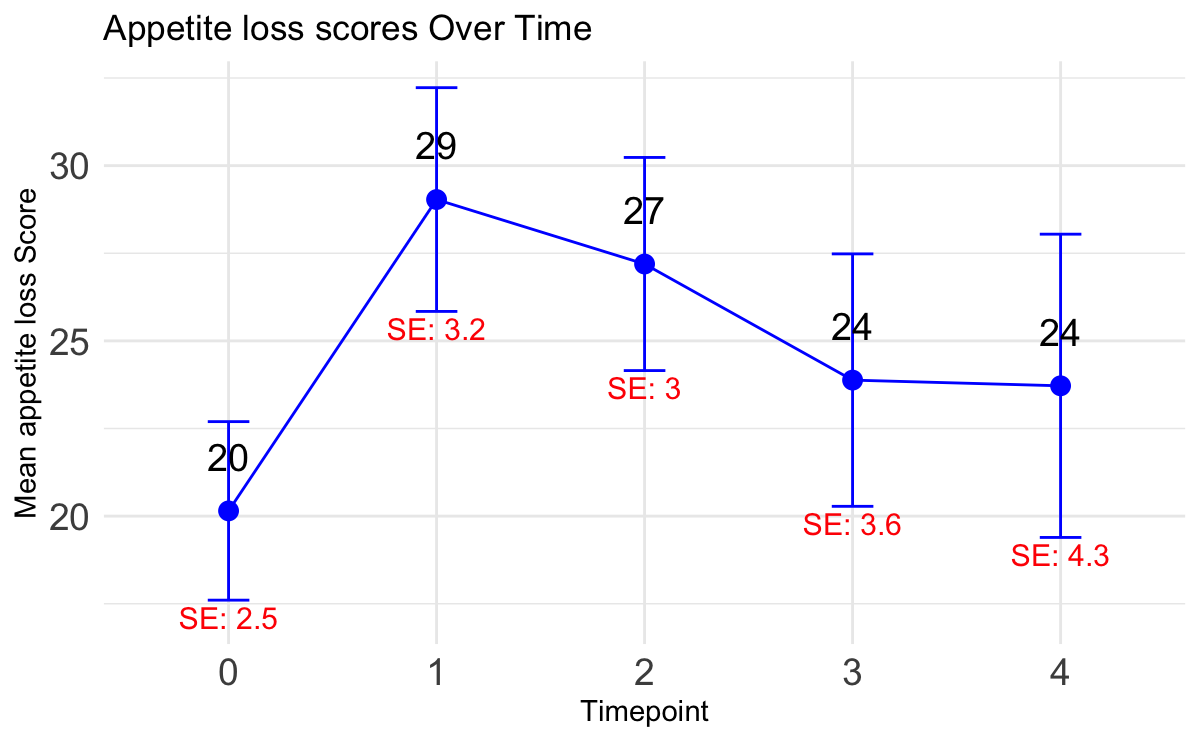


SE = standard errror

Mean loss of appetite scores for the 4 time points (T0, T1, T2, T3, T4) are represented on the y- axis. Mean loss of appetite score for T4 artificial is 23.7 (SE 4.3). The mean difference between T0 and T4 artificial is + 8.5 points (p=0.00982).

## Supplementary 4 : Descriptive statistics EORTC QLQ-C30 function domains

### Physical function


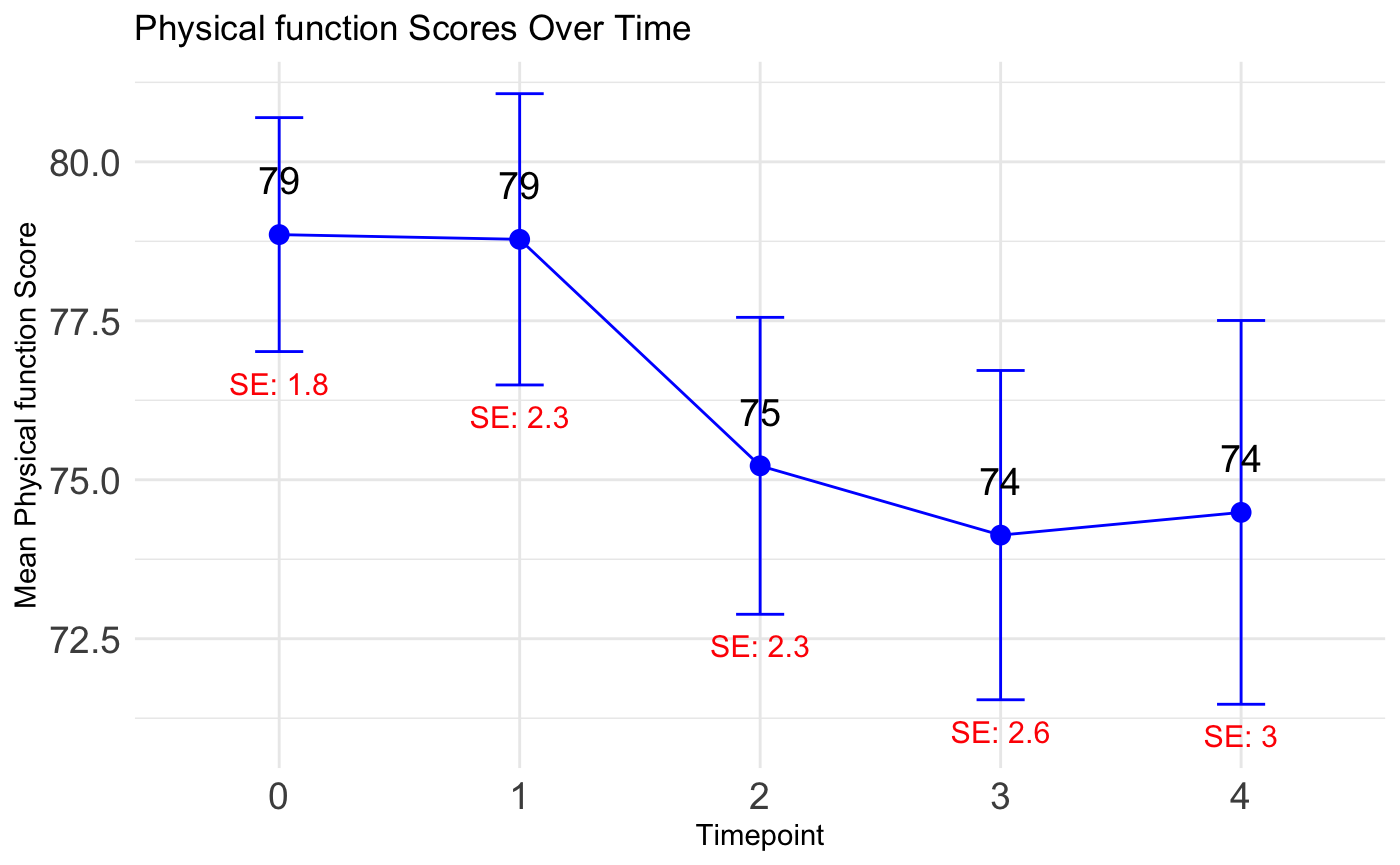


SE = standard errror

Physical function scores over time. Mean scores for the 4 time points (T0, T1, T2, T3, T4) are represented on the y- axis. Mean score for T4 artificial is 73.6 (SE 2.3). The mean difference between T0 and T4 artificial is -7.2 points (p<0.001).

### Cognitive function


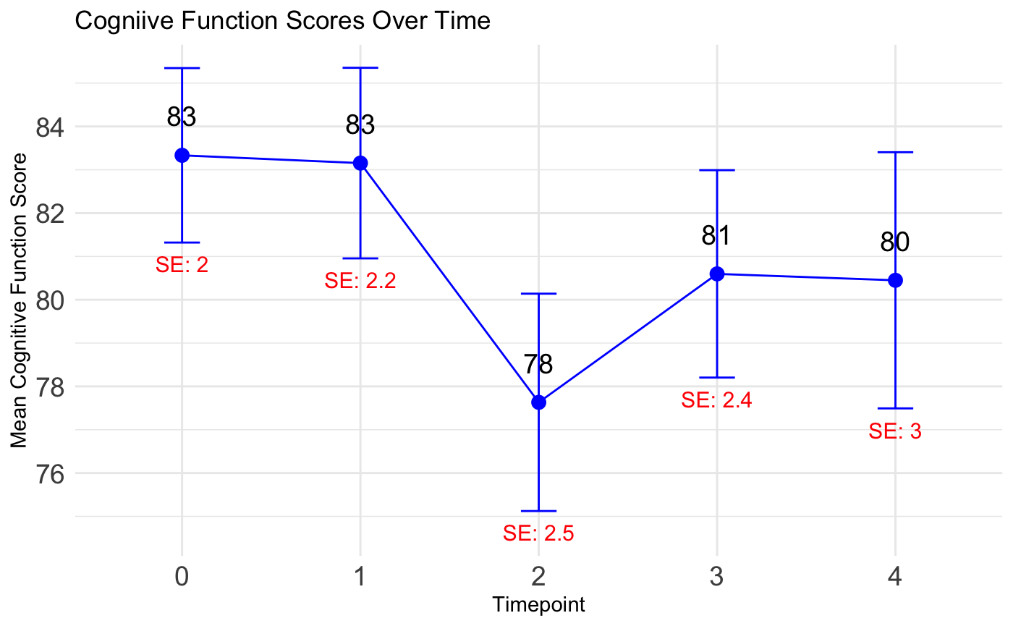


SE = standard errror

Cognitive function scores over. Mean scores for the 4 time points (T0, T1, T2, T3, T4) are represented on the y- axis. Mean score for T4 artificial is 79.3 (SE 2.2). The mean difference between T0 and T4 artificial is -6.0 points (p= 0.001549).

### Social function


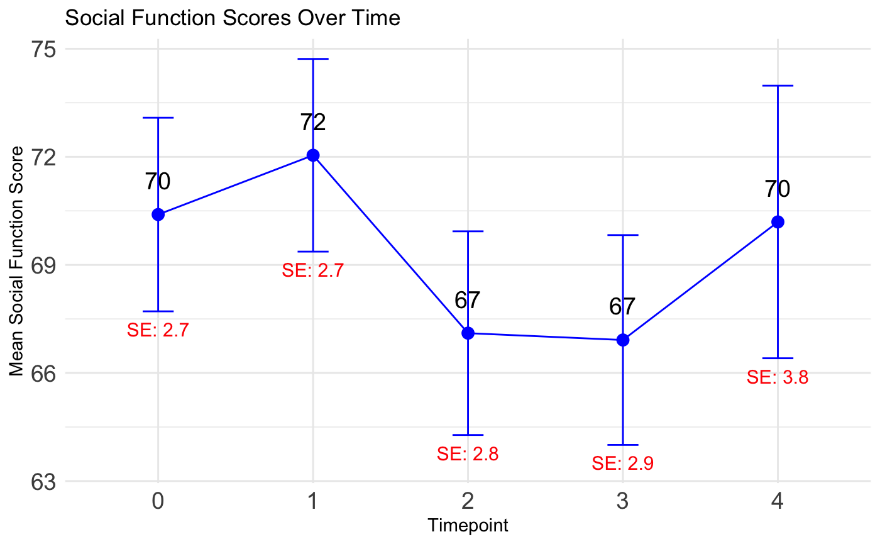


SE = standard errror

Social function scores over time. Mean scores for the 4 time points (T0, T1, T2, T3, T4) are represented on the y- axis. Mean score for T4 artificial is 66.1 (SE 2.8). The mean difference between T0 and T4 artificial is +5.6 points (p=0.07593).

### Role function


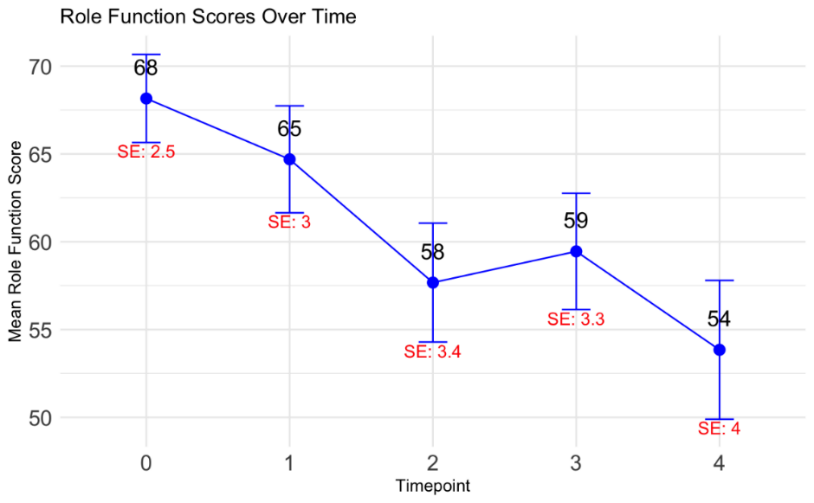


SE = standard errror

Role function scores over time. Mean scores for the 4 time points (T0, T1, T2, T3, T4) are represented on the y- axis. Mean score for T4 artificial is 53.7 (SE 3.0). The mean difference between T0 and T4 artificial is -17.2 points (p<0.001).

### Emotional function


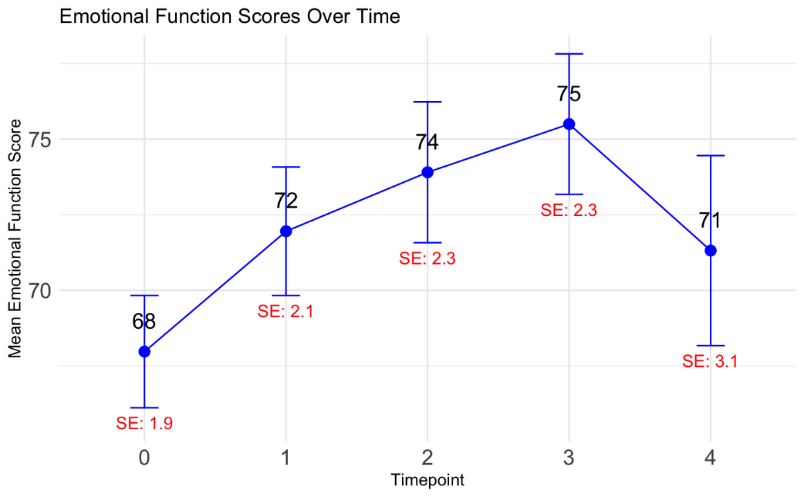


SE = standard errror

Emotional function scores over time. Mean scores for the 4 time points (T0, T1, T2, T3, T4) are represented on the y- axis. Mean score for T4 artificial is 71.7 (SE 2.2). The mean difference between T0 and T4 artificial is -0.8 points (p=0.7057).

## Supplementary 5 : Symptom domains: fatigue: univariate analysis (LME models)


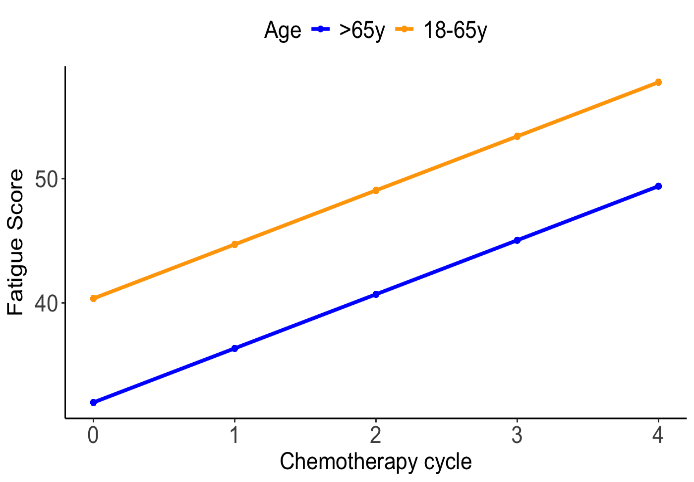

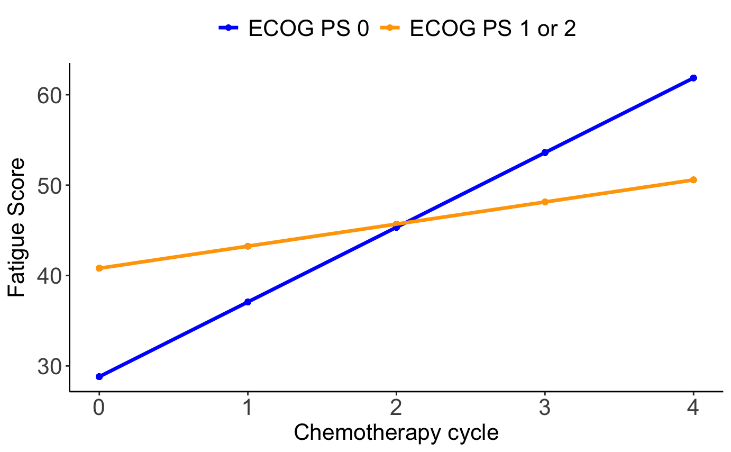


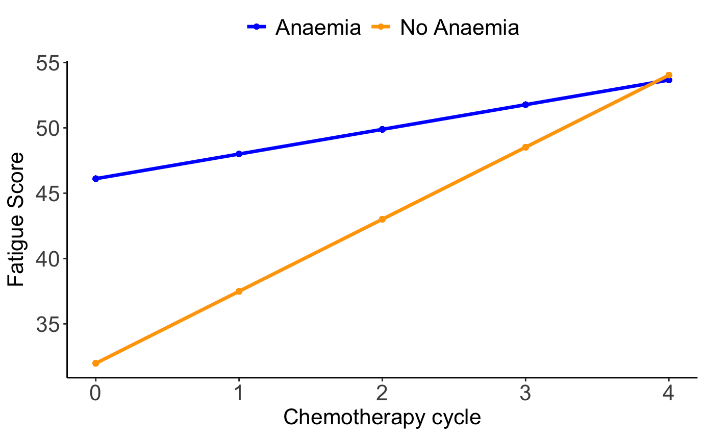

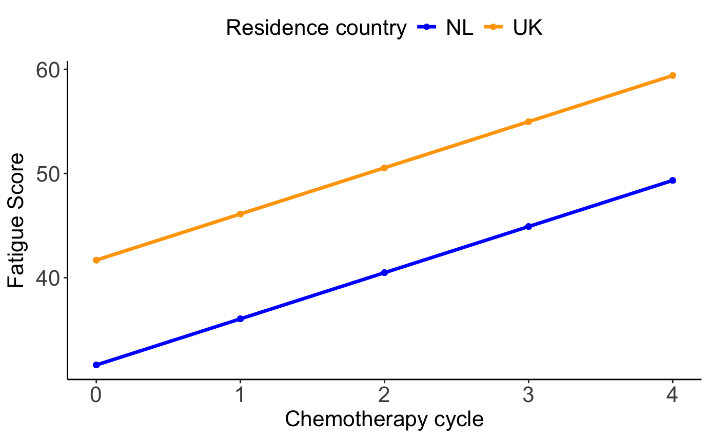


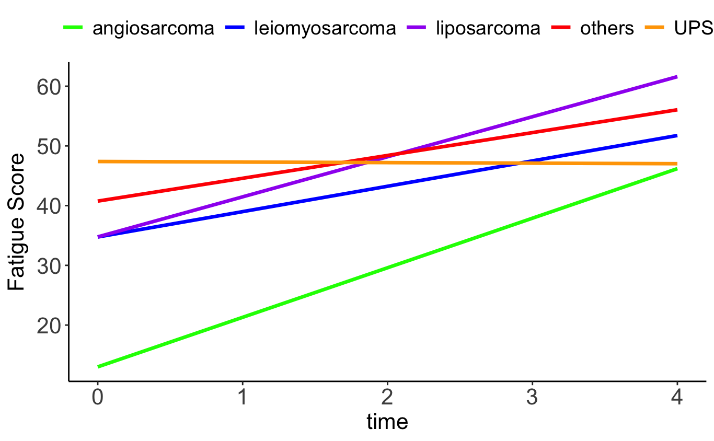


## Fatigue scores over time, using linear mixed effect models .A) Baseline fatigue scores are higher for patients aged 18-65 years (40.4) ( > 65 years 32.0, p = 0.035). No difference in the change rate over time between both groups (p=0.517). B) Higher baseline fatigue scores for patients with ECOG PS 1 or 2 (40.8 vs 28.8 [ECOG PS 0], p=0.012). Faster increase in fatigue scores over time in patients with ECOG PS 0 compared to those with ECOG PS 1-2 (change rate 8.3 vs 2.4 [ECOG PS 0], p<0.001). C) Higher baseline fatigue scores for patients with anaemia (46.1 vs 32.0 [no anaemia], p=0.002). Increase of fatigue scores over time for non-anemic patients compared to anemic patients (change rate 5.5 vs 1.9, p=0.029). D) Higher baseline fatigue scores in the UK ( 41.7 vs 31.6 [NL], p-value = 0.010). Increase of fatigue scores in NL and UK over time (p<0.05) No difference in the change rate over time between NL and UK (p=0.818). E) Lower baseline fatigue scores for patients with angiosarcoma compared to all other subgroups (p<0.05). Fatigue scores increase over time for all sarcoma subtypes (p<0.05), except for UPS ((change rate -0.4, p=0.874). No significant differences in change rates between the other sarcoma subtypes

## Supplementary 6 : Symptom domains: dyspnoea: univariate analysis (LME models)


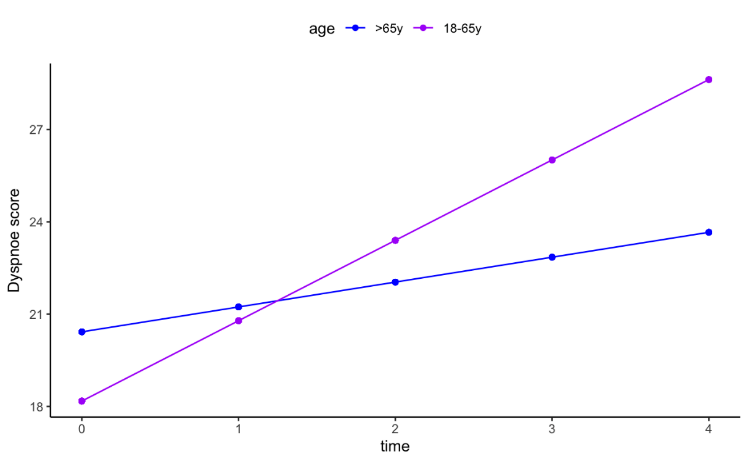


Over time there is an increase in dyspnoe scores for patients 18-65 years (change rate 2.6, p=0.0345) whereas scores for patients >65 years did not change over time (change rate 0.8, p=0.5581). There was no significant difference in scores at baseline (p=0.6132).


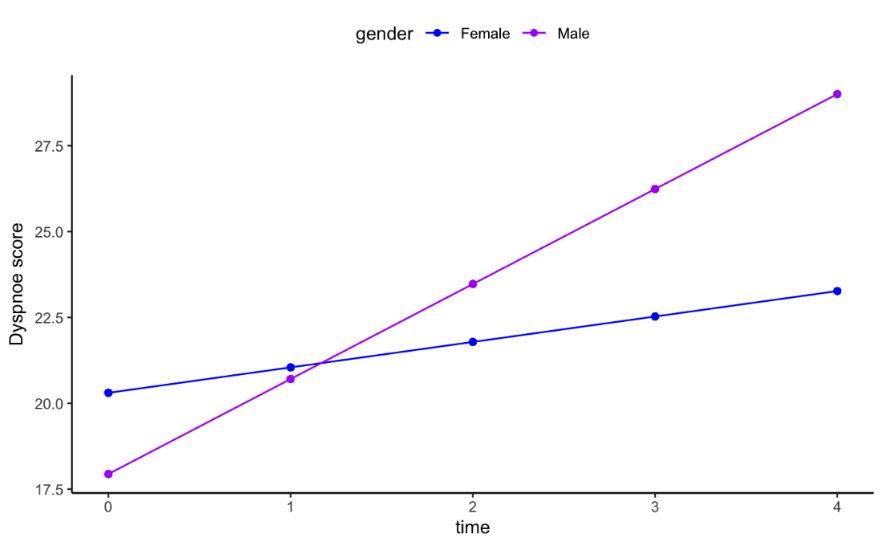


For male patients dyspnoe scores increase over time (change rate 2.8, p=0.0301) whereas for female patients scores remained stable over time (change rate 0.7, p=0.5803). There was no significant difference between scores at baseline (p=0.5897).


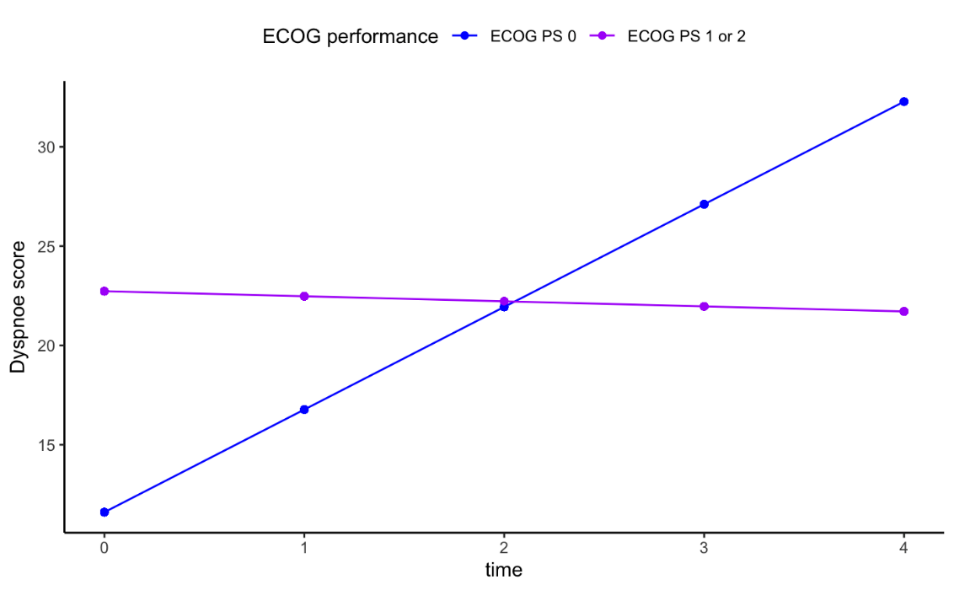


For patients with ECOG PS 0 dyspnoe scores increase over time (change rate 5.2, p=0.0011) whereas for patiens with ECOG PS 1 or 2 dyspnoe scores remain stable over time (change rate -0.3, p=0.8123). At baseline, dyspnoe scores are significantly higher for patients with ECOG PS 1 or 2 (22.7 vs 11.6 p=0.0135).


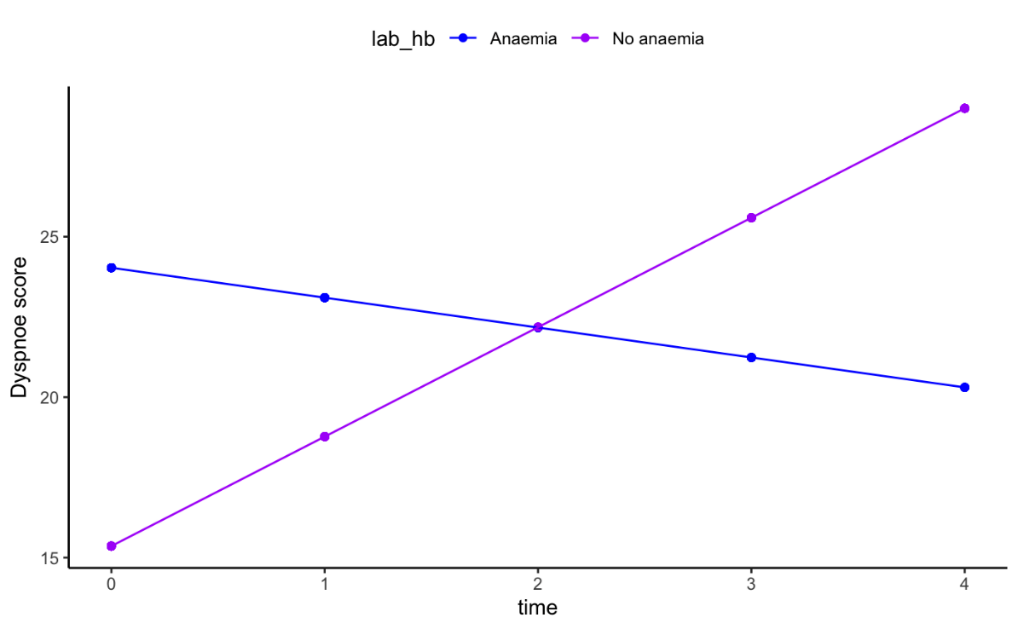


For patients with normal hemoglobin at baseline there is an increase of dyspnoea scores over time (change rate +3.4, p=0.0016) whereas for patients with anemia at baseline there is no significant change over time of dyspnoea scores (change rate -0.9, p=0.5682). There is no significant difference in dyspnoea scores at baseline between both groups (p=0.0542).


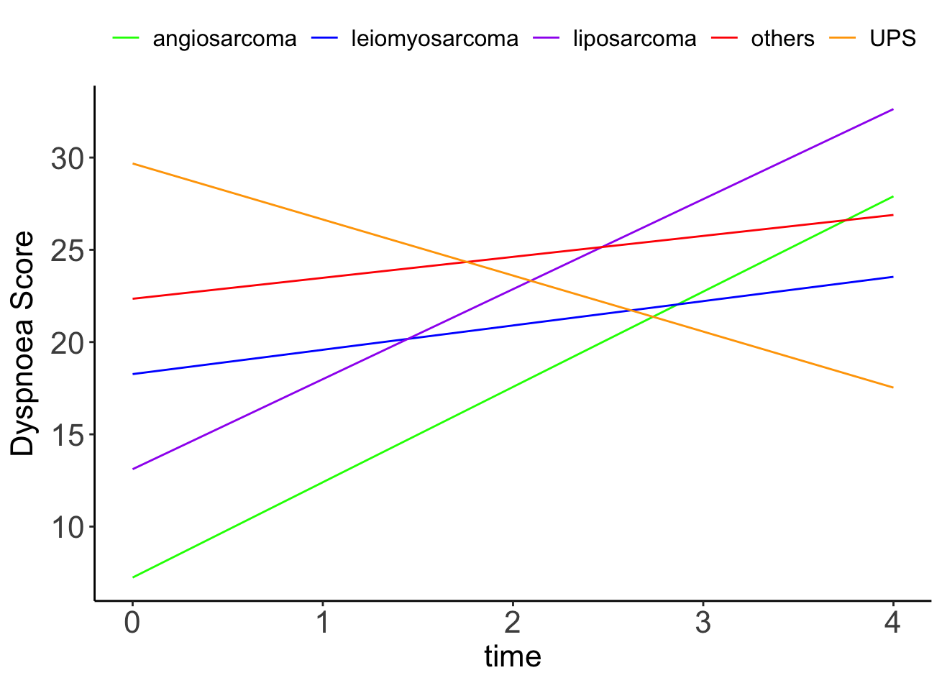


Patients with UPS had worse baseline scores (29.7) than those with liposarcoma (13.1, p=0.0316) and angiosarcoma (7.2, p=0.0391); no baseline differences in other subgroups (p>0.05). Dyspnoea scores worsened over time in angiosarcoma (+5.9) and liposarcoma (+4.9) patients, with no change in leiomyosarcoma, UPS, or the subgroup ‘other’ (p>0.05).

## Supplementary 7 : Symptom domains: appetite loss: univariate analysis (LME models)

**ECOG**

**
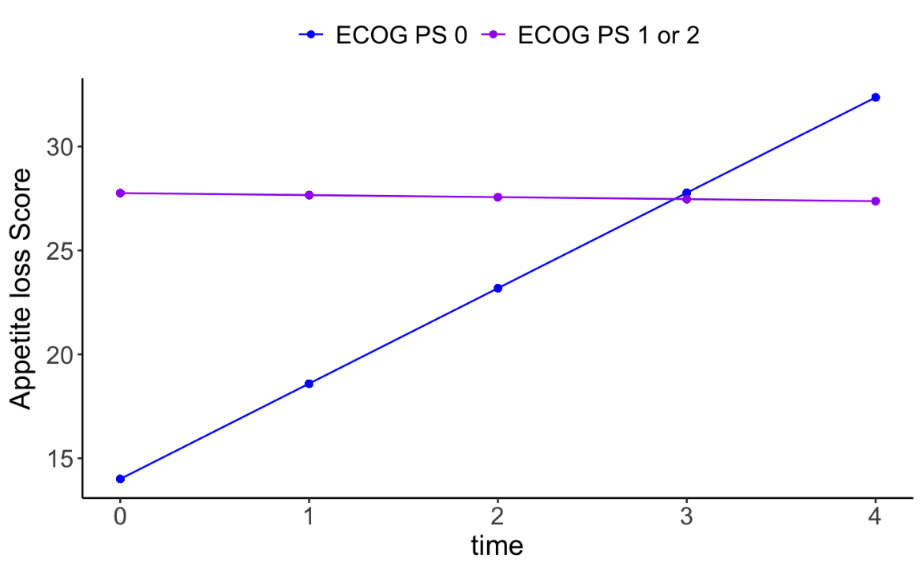
**

Worse baseline appetite loss scores in patients with ECOG PS 1-2 (27.8 vs 14.0 [ECOG PS 0], p=0.0083). Only change over time (i.e. worsening) in patients with ECOG PS 1-2 (change rate 4.6, p=0.0278)


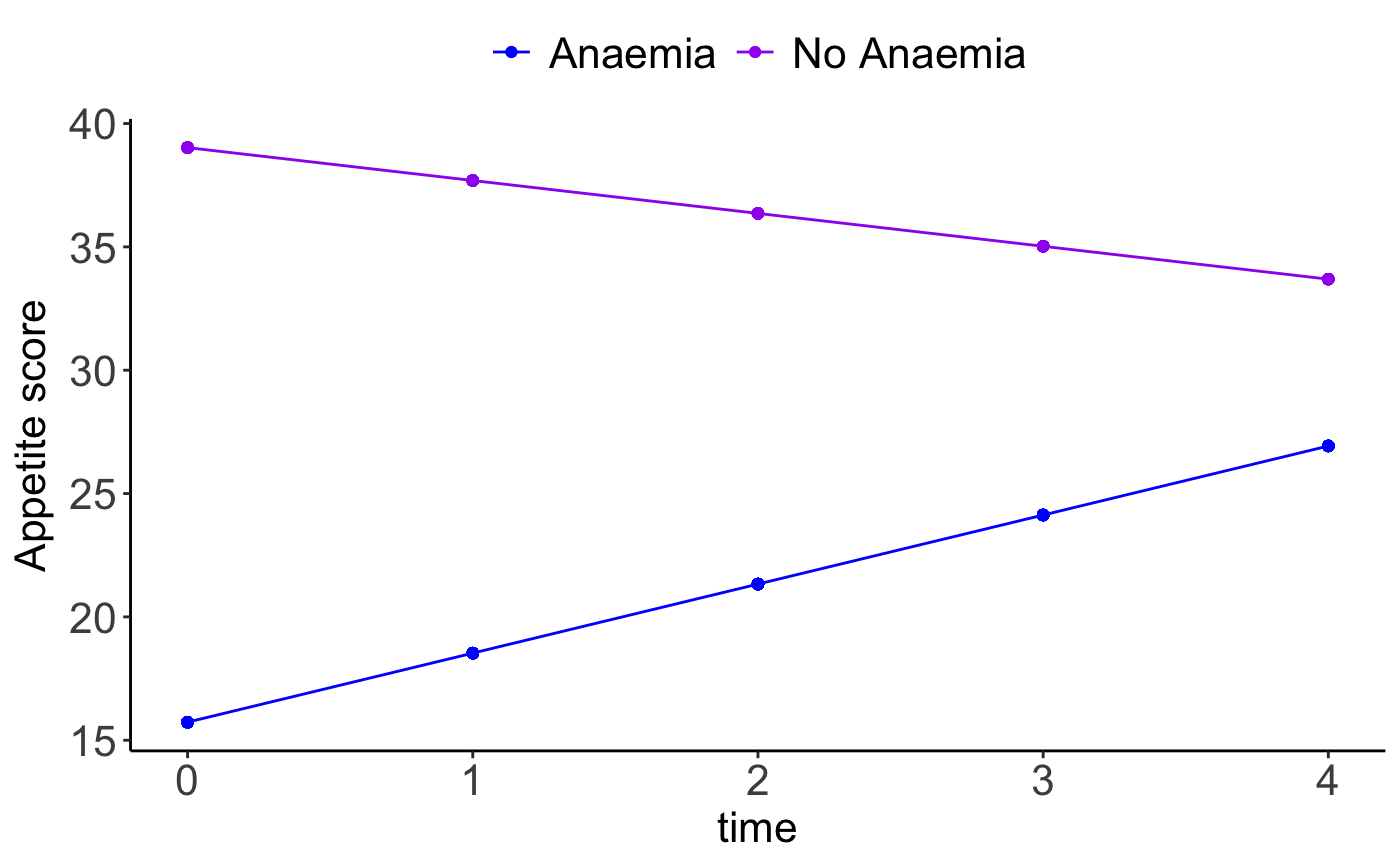


Worse baseline appetite loss scores in patients with anaemia (39.0, vs 15.7 [anaemia], p <0.001). Decrease of appetite loss scores over time in the first group (change rate -1.3) compared to an increase over time in the latter group (change rate +2.8, p 0.0382).

## Supplementary 8 : Symptom domains: constipation: univariate analysis (LME models)


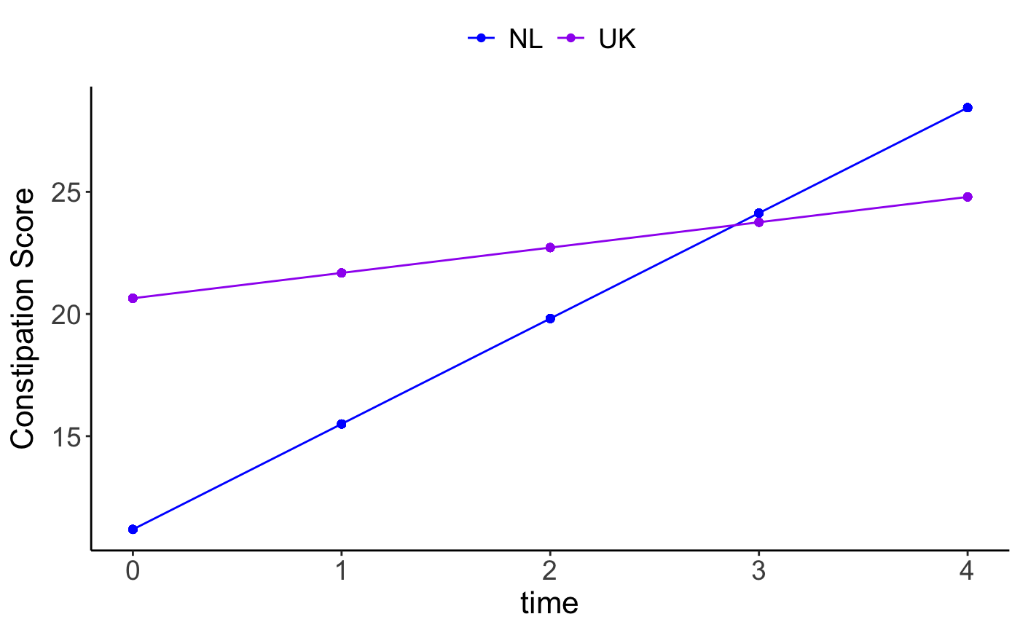


Baseline constipation scores are worse in UK compared to NL patients (20.6 vs 11.18, p= 0.0329). Over time. There was a tendency towards a faster worsening of scores in NL patients (change rate 1.0 vs 4.3, p=0.0540).


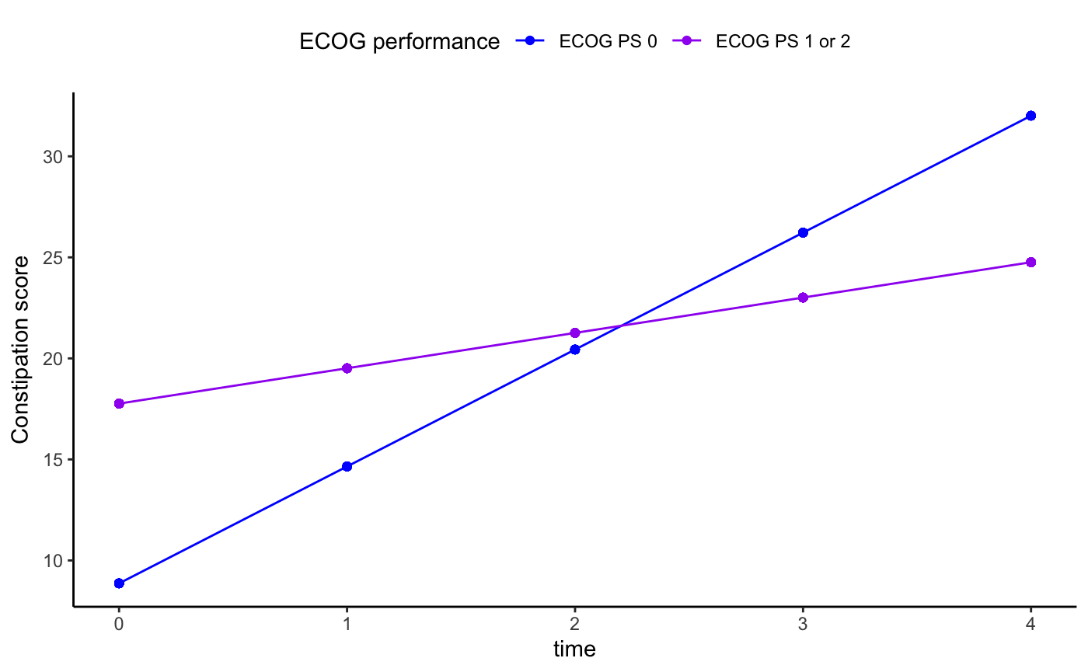


Patients with ECOG PS 0 have a tendency towards lower baseline constipation scores (8.9 vs 17.8, p =0.0521) but experience a sharper increase in constipation scores compared to patients with ECOG PS 1-2 (change rate 5.8 vs 1.7, p=0.0338).


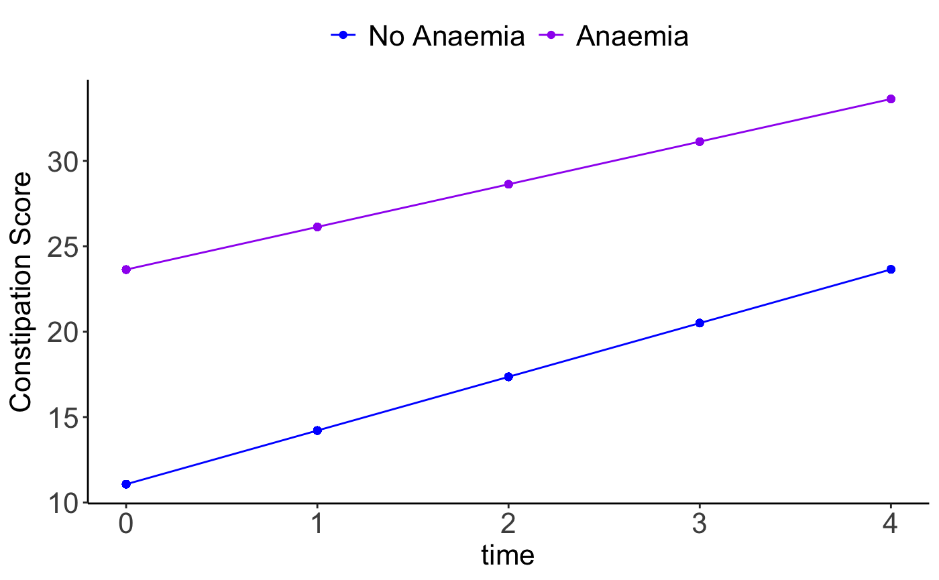


Worse constipation scores at baseline in patients with anaemia compared to patients without anaemia (23.1 vs 11.3, p=0.0035). No difference in the change rate over time.

## Supplementary 9 : Symptom domains: nausea and vomiting : univariate analysis (LME models)


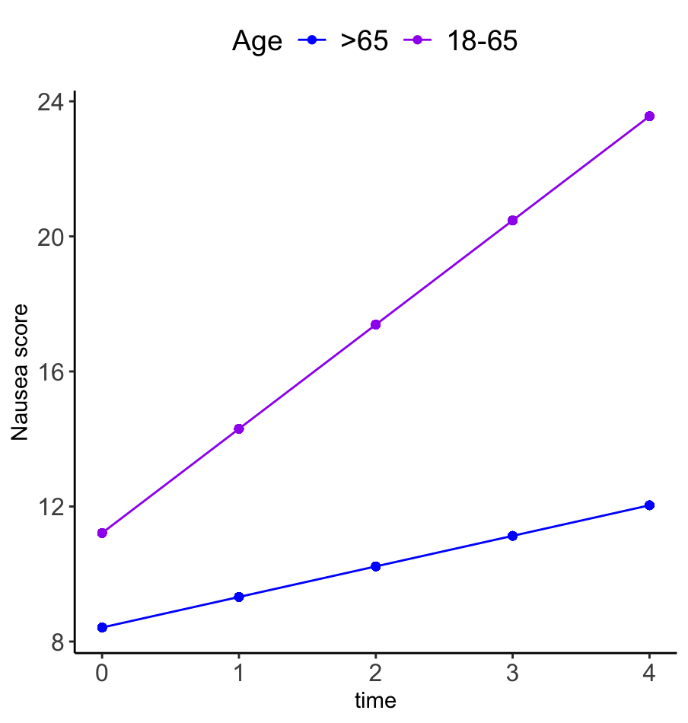


No significant difference in baseline nausea and vomiting scores between both groups (p=0.3187). Sharper increase (i.e. worsening) of scores in patients aged 18-65 years (change rate + 3.1 vs 0.9, p=0.0481.


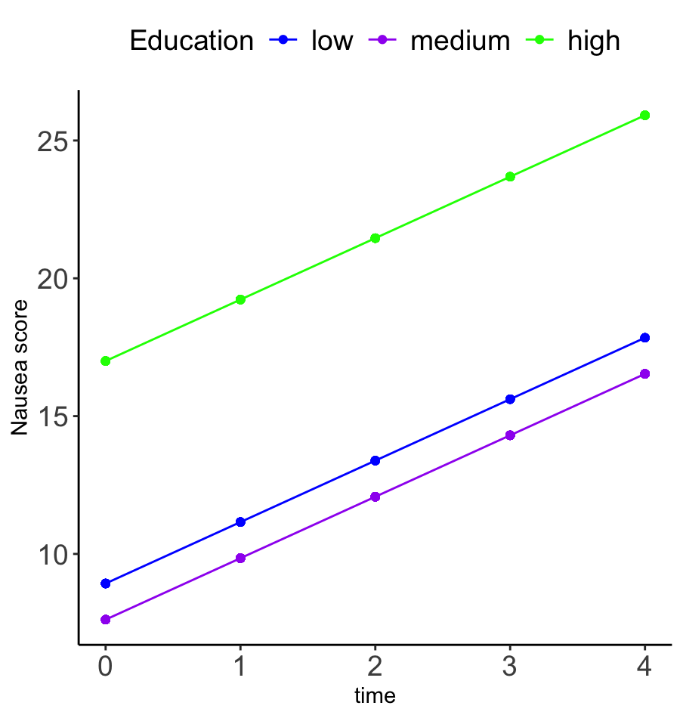


Worse baseline nausea and vomiting scores in patients with high educational level (17.0) compared to medium (7.6, p 0.0044) and low (8.9, p 0.0488). No differences in change rates over time (p>0.05).

**
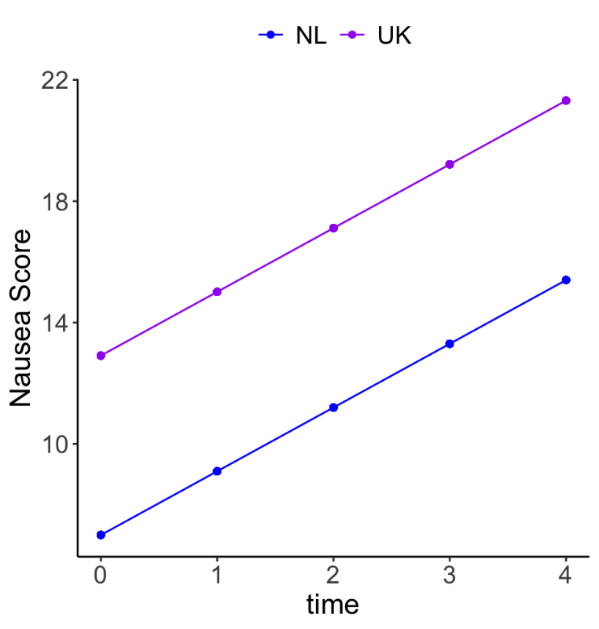
**

Worse baseline nausea and vomiting scores in UK patients (12.9) compared to NL patients (7.0, p 0.0240). No differences in change rates over time (p>0.05).


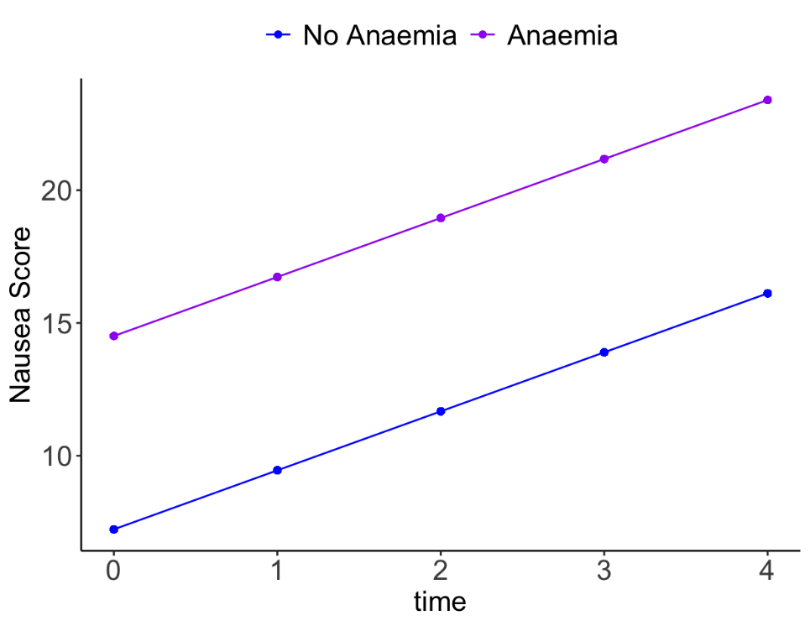


Patients with anaemia have higher baseline nausea and vomiting scores (14.5 vs 7.2, p=0.0063). No significant differences in change rates over time (p>0.05).


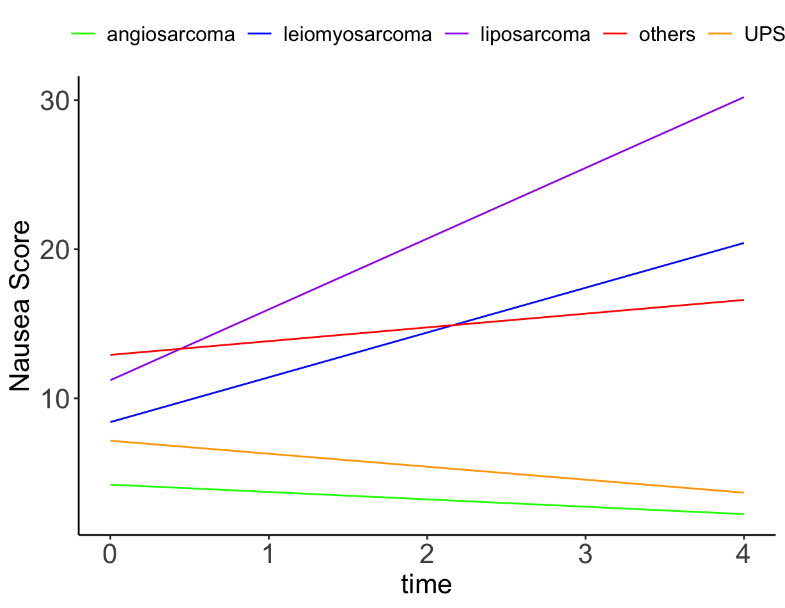


Faster increase in scores in patients with liposarcoma (change rate + 4.7, p<0.001) and LMS (change rate +3.0, p=0.0037). Scores remain stable in patients with UPS (p 0.5769),  angiosarcoma (p 0.7951) and the subgroup 'other' (p=0.3245).

## Supplementary 10 : Symptom domains: insomnia: univariate analysis (LME models)


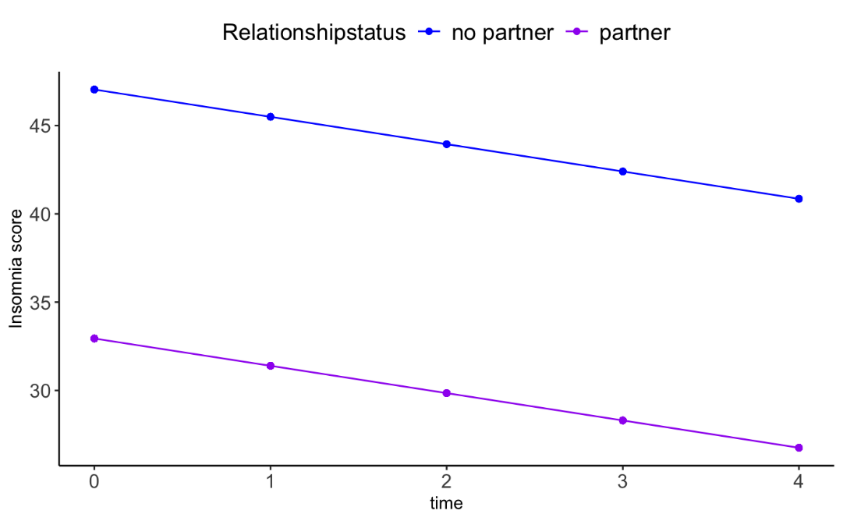


Worse baseline insomnia scores in patients without a partner (47.0 vs 32.9 , p=0.0161). No difference in the change rate over time between subgroups (p>0.05).


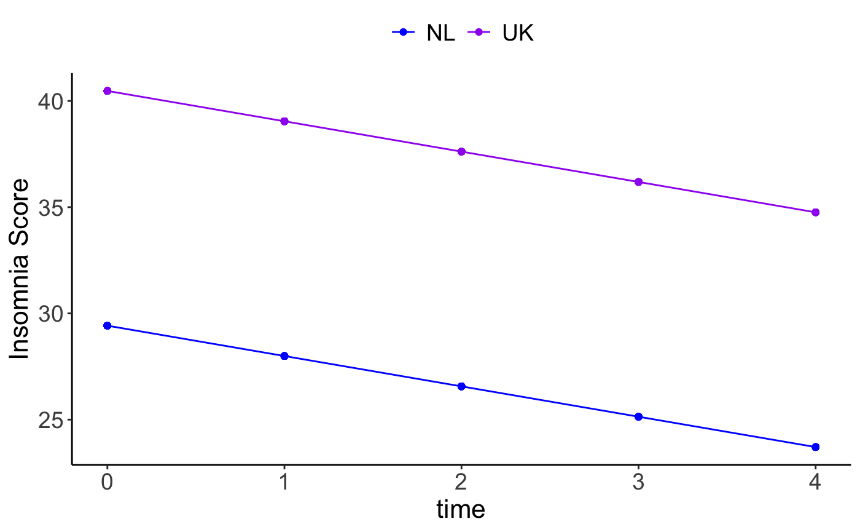


Worse baseline scores for UK patients compared to NL patients (40.5 vs 29.4, p=0.1268). No significant difference in the change rate over time. No difference in the change rate over time between groups (p>0.05).


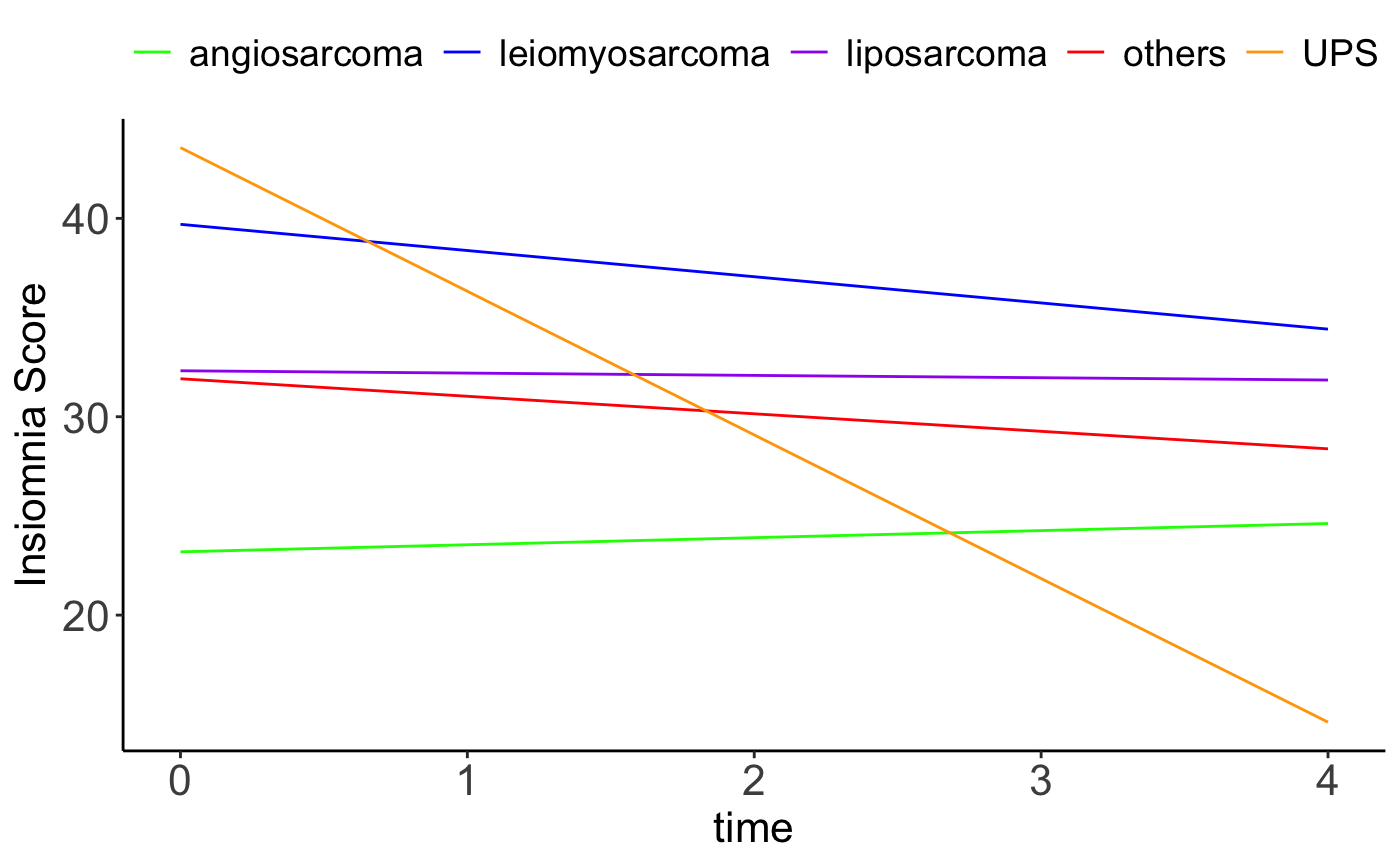


No differences in insomnia scores between subgroups at baseline (p>0.05). No change over time, except for the subgroup with UPS (change rate -7.2, p=0.0091).


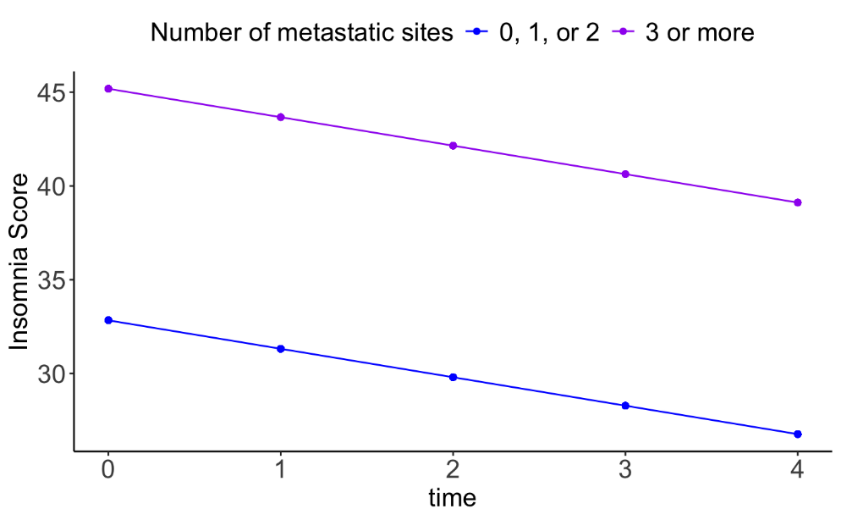


Tendency towards worse baseline insomnia scores in patients with >2 metastatic sites compared to those with ≤2 metastatic sites (44.9 vs 32.9, p=0.0p<587). No change over time (p>0.05).

## Supplementary 11 : function domains: physical function : univariate analysis (LME models)


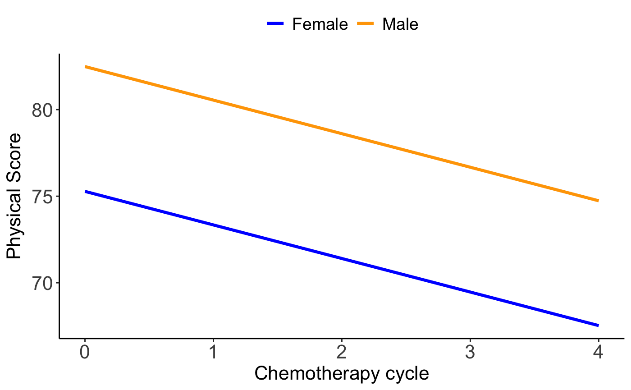

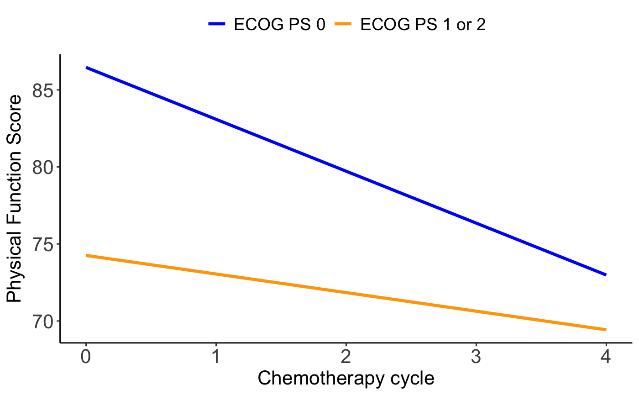


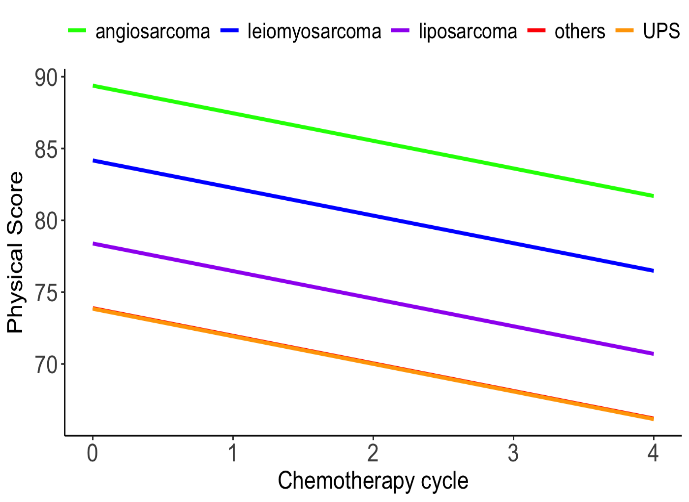


**Physical function scores over time, using linear mixed effect models.** **A)** Higher (i.e.) better baseline physical function scores in male patients compared to females (82.5 vs 75.3, p= 0.036). Scores decrease over time in both groups without a difference in the change rate (p>0.05). **B)** Higher baseline physical function scores in patients with ECOG PS 0 (86.5 vs 74.3, p=0.002) but sharper decrease over time (change rate -3.4 vs -2.2, p=0.022). **C)** Lower baseline physical function scores in the subgroup other (73.9) compared to angiosarcoma (89.4, p=0.0427) and leiomyosarcoma (84.2, p=0.022). No differences in change rates over time (p>0.05).

## Supplementary 12 : function domains: social function : univariate analysis (LME models)


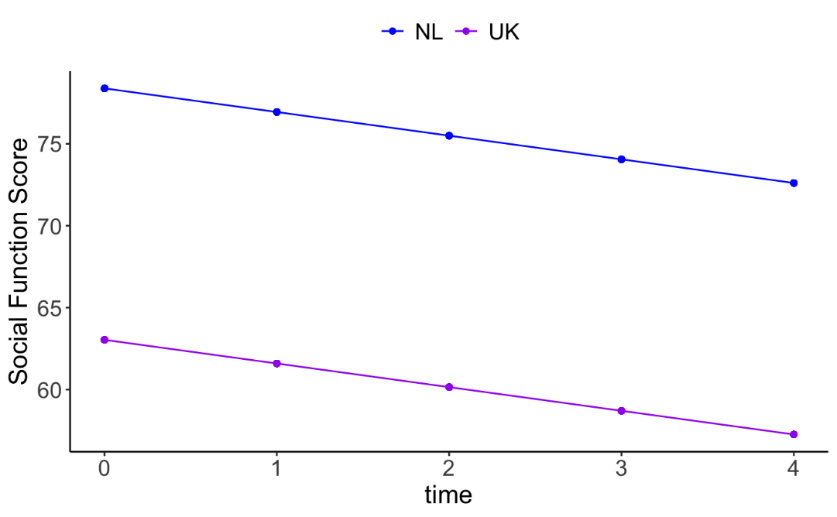


Higher baseline social function score in NL patients compared to UK (78.4 vs 63.0, p=0.0001). No change in scores over time (p>0.05).


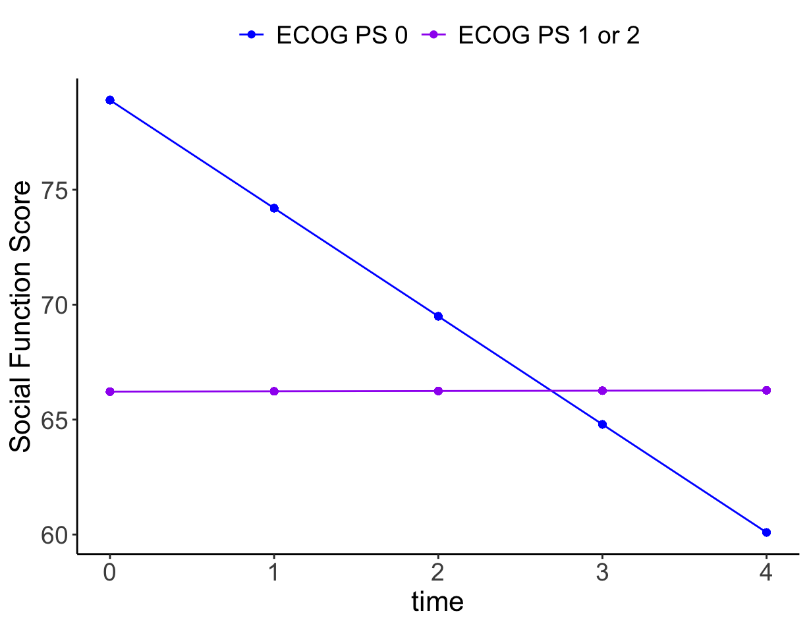


Higher baseline social function scores in patients with ECOG PS 0 compared to ECOG PS 1-2 (78.9 vs 66.2, p=0.0169) but sharper decrease (i.e. worsening) in patients with ECOG POS 0 (change rate -4.70193 vs +0.01341, p=0.0079).


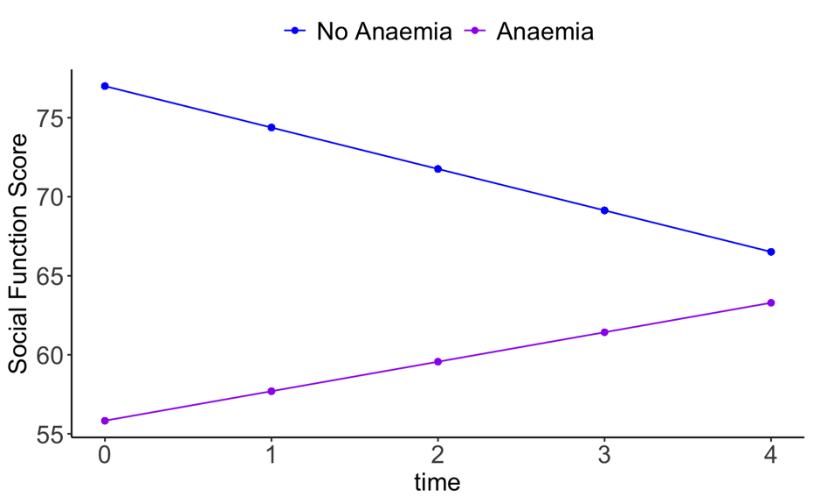


Higher baseline social function scores in patients without anaemia compared to those with anaemia (77.0 vs 55.8, p<0.001). Over time increase (i.e. improvement) of scores in patients with anaemia (change rate +1.9) compared to a decrease over time in patients without anaemia (change rate -2.6, p= 0.0083).

## Supplementary 13 : function domains: role function : univariate analysis (LME models)


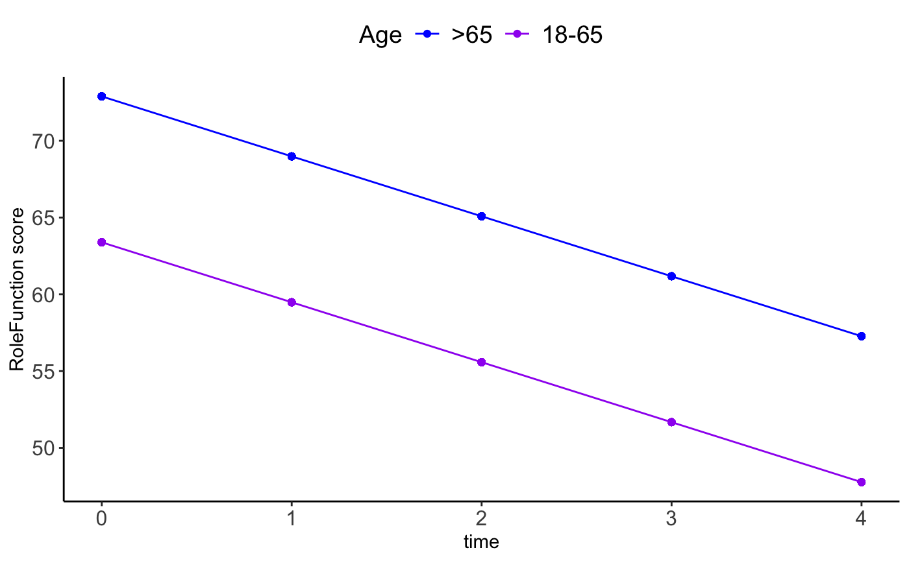


Higher baseline role function scores in patients > 65 compared to patients aged 18-65 (72.8 vs 63.4, p=0.0233). No difference in the change rate over time (p>0.05).


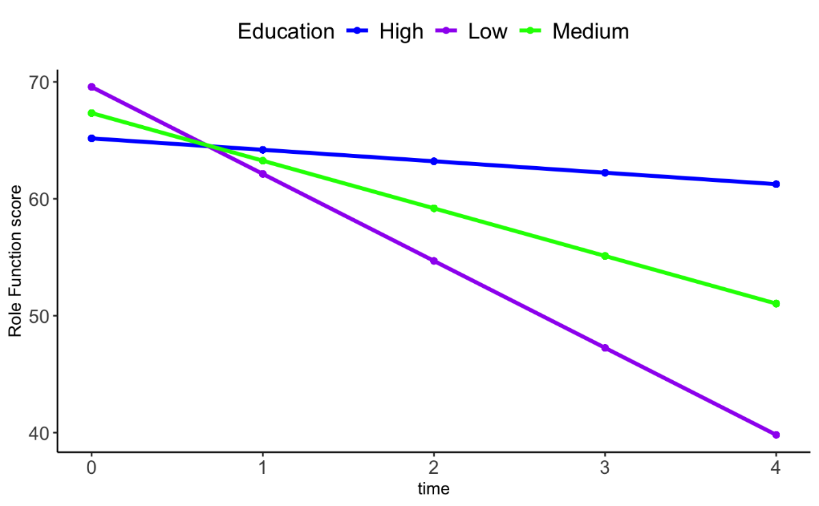


Stable role function scores for patients with a high educational level (change rate -1.0, p=0.5456). Decrease over time in patients with low (change rate -7.4) and medium (change rate -4.07378).


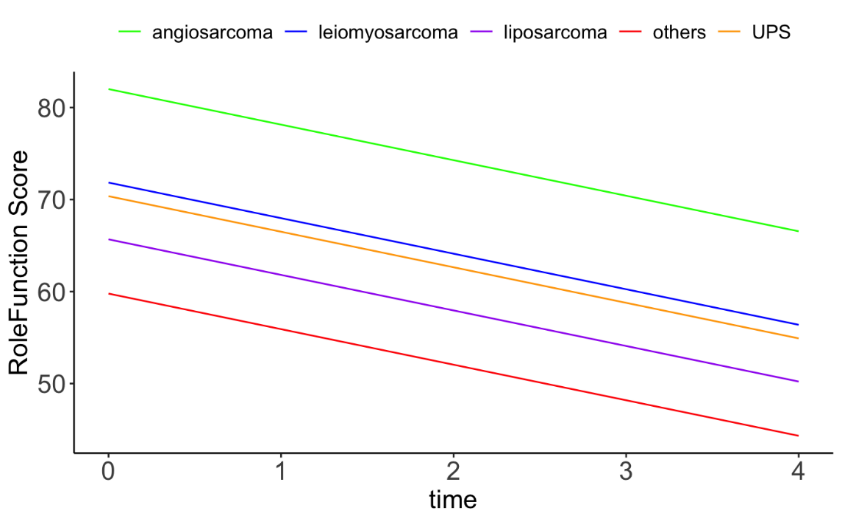


Lower baseline role function scores in the subgroup 'other' (59.77)  compared to liposarcoma (71.8, p=0.2942) and angiosarcoma (82.0, p=0.0148). No differences at baseline or over time (p>0.05).


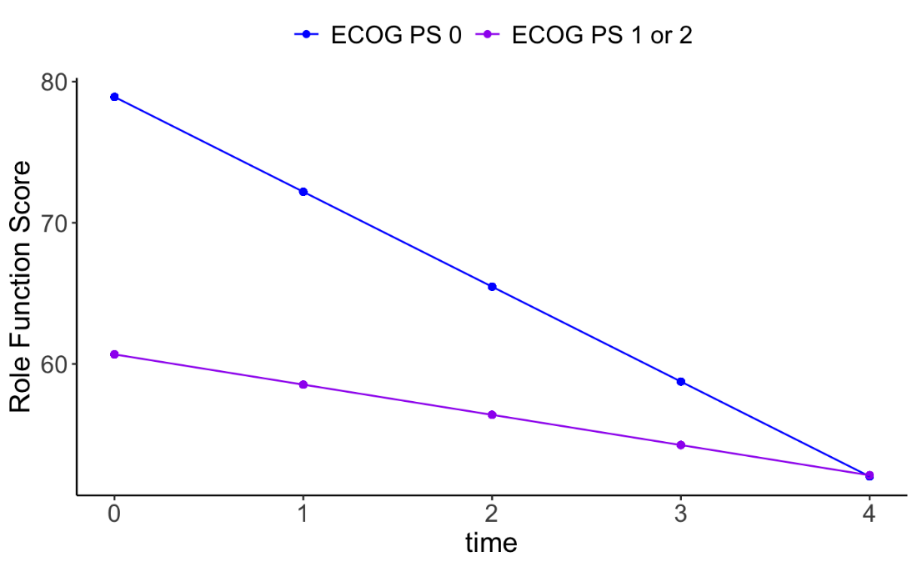


Higher baseline role function scores in patients with ECOG PS 0 compare to those with ECOG PS 1-2 (78.9 vs 60.7 p=0.0002). Sharper decrease in scores in patients with ECOG PS 0 (change rate -6.7 vs -2.1, p=0.0119).


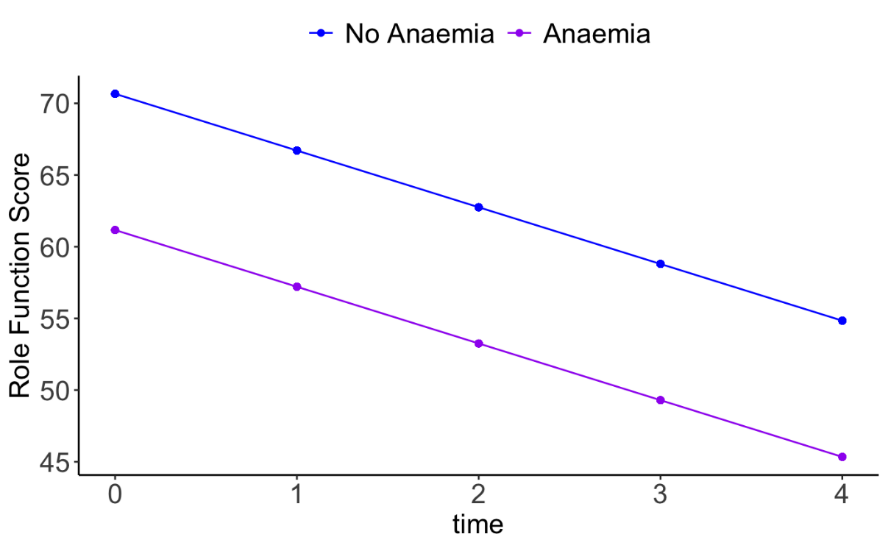


Higher baseline role function scores in patients without anaemia (70.7 vs 61.2, p=0.0308). No difference in change rates over time between both groups (p>0.05).

## Supplementary 14: function domains: emotional function: univariate analysis (LME models)


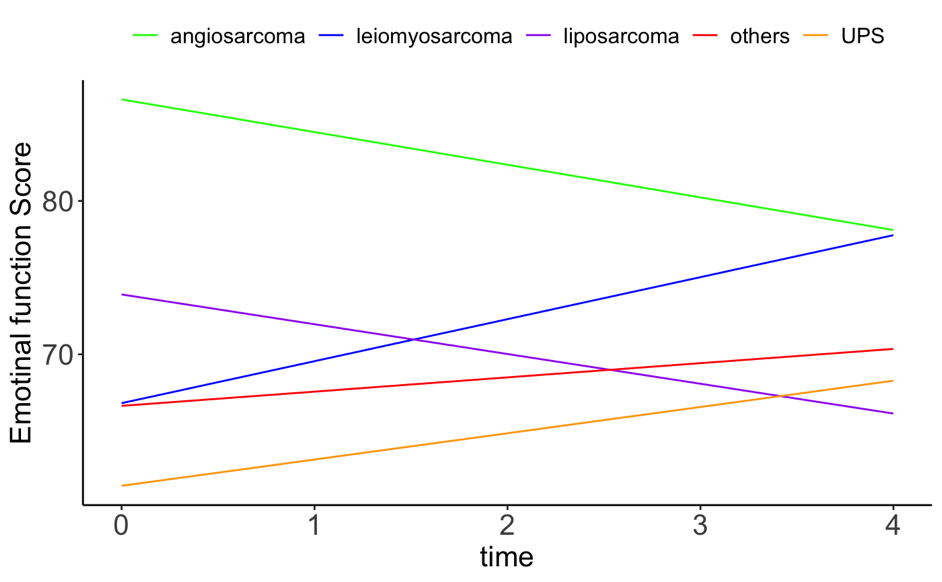


Lower baseline emotional function scores in UPS (61.4, p= 0.0028) and STS subtype ‘other’ (66.6, p= 0.0083) compared to angiosarcoma (86.6) and lower baseline score for UPS (61.4) compared to liposarcoma (73.9, p= 0.0353). Decrease over time for angiosarcoma (change rate -2.1) compared to an increase over time in leiomyosarcoma (change rate, +2.7). Decrease over time for liposarcoma (change rate -1.9) compared to an increase over time of scores for leiomyosarcoma (change rate + 2.7, p= 0.0032) .

## Supplementary 15: function domains: cognitive function: univariate analysis (LME models)


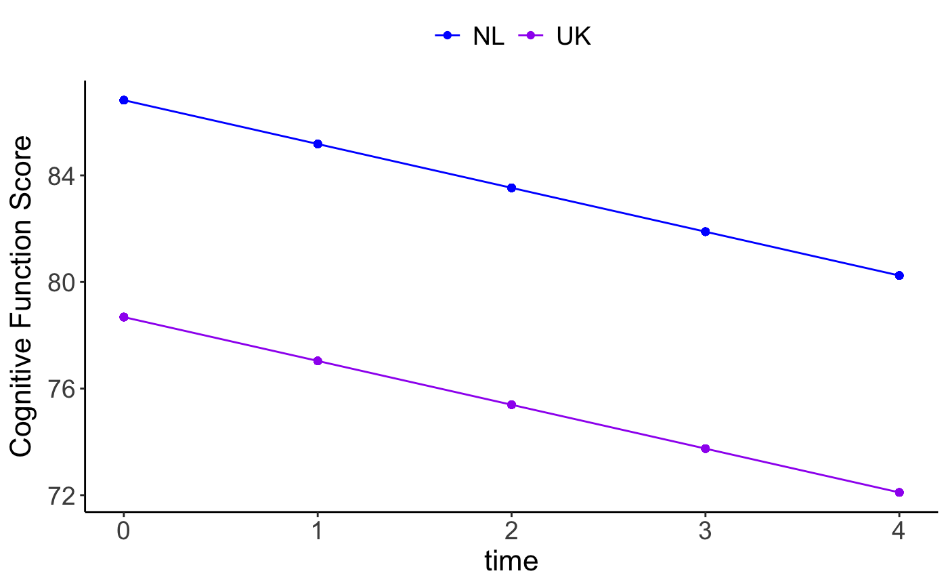


Higher baseline cognitive function scores in NL patients (86.8 vs 78.7 [UK]). No difference in the change rate over time (p>0.05).


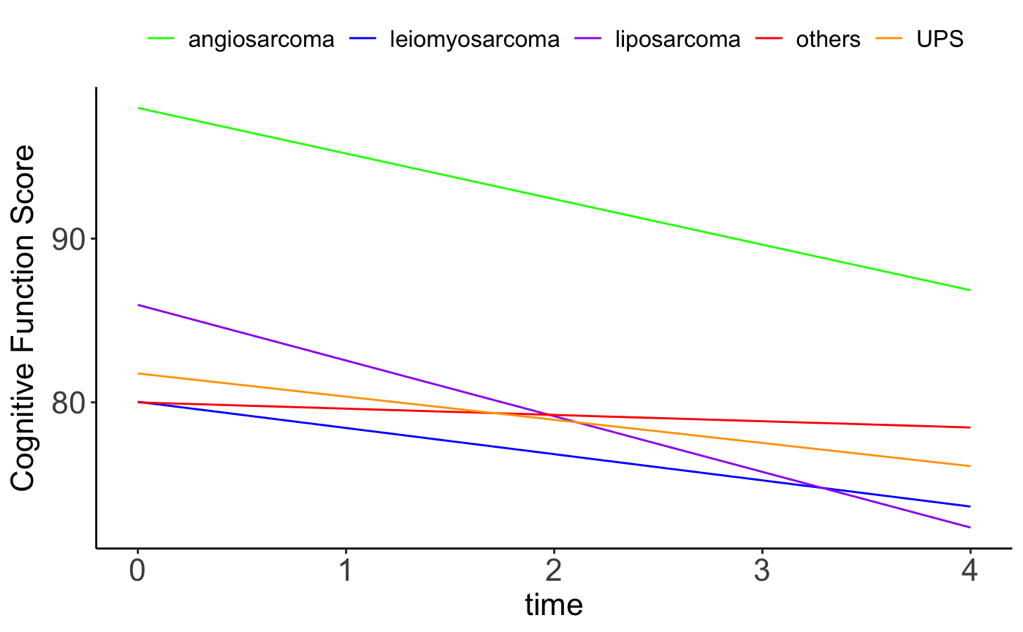


Higher baseline cognitive function scores in angiosarcoma (98.0) compared to the STS subgroup ‘others’ (80.0, p=0.0374) and to leiomyosarcoma (90.0, p= 0.0378). No change over time for angiosarcoma (change rate -0.4, p= 0.6626) and ‘others’ (change rate -2.8, p= 0.1229), while there is a decrease over time for the other subgroups (p<0.05).


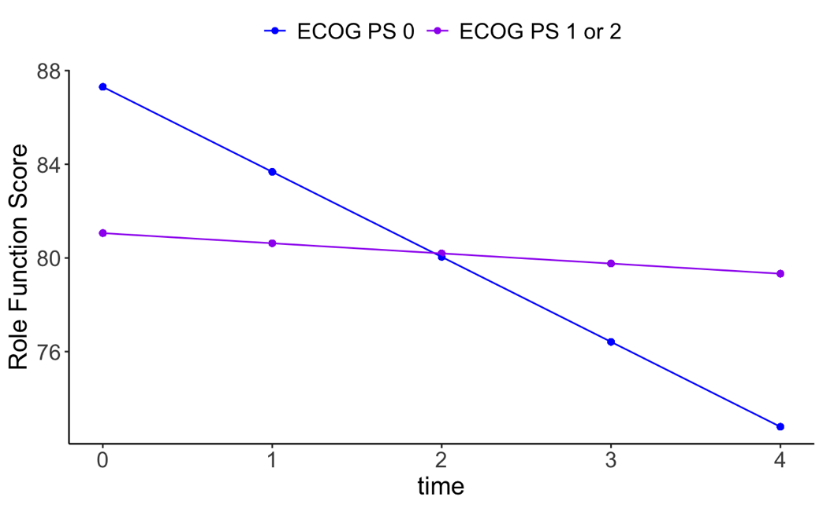


No differences in baseline role function score between both groups (p= 0.1209). Faster decrease of scores in patients with ECOG PS 0 compared to ECOG PS 1-2 (change rate -3.6 vs -0.4, 0.0052).


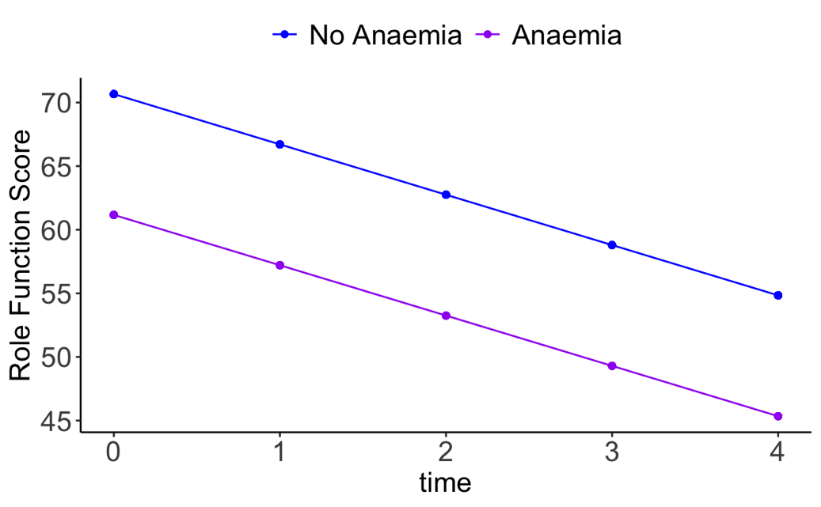


Higher baseline role function scores in patients without anaemia compared to patients with anaemia (70.7 vs 61.2, p= 0.0308). No difference in the change rate between both groups (p>0.05).
